# Assessment of Depression and Adherence to Guideline-Directed Medical Therapies Following Percutaneous Coronary Intervention

**DOI:** 10.1001/jamanetworkopen.2022.46317

**Published:** 2022-12-12

**Authors:** Matthew E. Lapa, Gretchen M. Swabe, Bruce L. Rollman, Matthew F. Muldoon, Rebecca C. Thurston, Jared W. Magnani

**Affiliations:** 1 University of Pittsburgh School of Medicine, Pittsburgh, Pennsylvania; 2 Center for Behavioral Health and Smart Technology, Division of General Internal Medicine, University of Pittsburgh School of Medicine, Pittsburgh, Pennsylvania; 3 Department of Epidemiology, Graduate School of Public Health University of Pittsburgh, Pittsburgh, Pennsylvania; 4 Center for Research on Health Care, Department of Medicine, University of Pittsburgh, Pittsburgh, Pennsylvania

## Abstract

**Importance:**

Depression is associated with increased risk of primary and secondary cardiovascular events. Medication adherence may play an essential role.

**Objective:**

To evaluate the association of depression and 12-month adherence to guideline-directed medical therapies (eg, antiplatelet agents, β-blockers, renin-angiotensin-aldosterone system inhibitors [ie, angiotensin-converting enzyme inhibitors and angiotensin receptor blockers], and statins) following percutaneous coronary intervention.

**Design, Setting, and Participants:**

This retrospective cohort study included individuals who underwent percutaneous coronary intervention from January 1, 2014, to December 31, 2019. Data were collected from a large US health claims database and analyzed between February and August 2022.

**Main Outcomes and Measures:**

Proportion of days covered (PDC) for classes of guideline-directed medical therapies, with 12-month adherence categorized as adequate (PDC ≥80% to <90%) or optimal (PDC ≥90%). Multivariable-adjusted regression models were used to evaluate the association of depression with adherence; models incorporated demographic characteristics, comorbid medical and psychiatric conditions, depression treatment, and guideline-directed medical therapy treatment adjustment. The hypothesis was that those with depression would have lower odds of either adequate or optimal adherence to agents essential for guideline-directed medical therapy.

**Results:**

Of 124 443 individuals (mean [SD] age, 69.3 [10.6] years; 41 430 [33.3%] female sex; 3694 [3.0%] Asian, 12 611 [10.1%] Black, and 12 337 [9.9%] Hispanic individuals) who received percutaneous coronary interventions, 20 711 (16.6%) had a diagnosis of depression. Those with depression were significantly less likely to obtain adequate 12-month adherence to antiplatelets (odds ratio [OR], 0.80; 95% CI, 0.77-0.85), β-blockers (OR, 0.84; 95% CI, 0.80-0.88), and statins (OR, 0.88; 95% CI, 0.85-0.93) than those without depression; there was no association between depression and adherence to renin-angiotensin-aldosterone system inhibitors (OR, 0.93; 95% CI, 0.85-1.00). Those with depression had similarly decreased likelihood of optimal 12-month adherence to antiplatelets, β-blockers, and statins as well as renin-angiotensin-aldosterone system inhibitors (OR, 0.87; 95% CI, 0.82-0.94).

**Conclusions and Relevance:**

In this cohort study, patients with depression were less likely to achieve adequate or optimal adherence to medications essential to guideline-directed medical therapies following percutaneous coronary intervention compared with those without depression. Recognition of depression may facilitate targeted interventions to address medication adherence and thereby improve secondary cardiovascular disease prevention.

## Key Points

**Question:**

What is the association between depression and adherence to guideline-directed medical therapies following percutaneous coronary intervention?

**Findings:**

In this cohort study of 124 443 individuals who underwent percutaneous coronary intervention, those with depression were approximately 10% to 20% less likely to achieve 12-month optimal adherence to guideline-directed medical therapies than those without depression. These results persisted following adjustment for demographic characteristics, comorbid medical and psychiatric conditions, social factors (education and income), and medication adjustment.

**Meaning:**

These findings suggest that screening for and addressing depression in those with cardiovascular disease are essential for developing targeted strategies to improve medication adherence and enhance secondary cardiovascular prevention in individuals with depression.

## Teaser

This cohort study evaluates the association of depression with 12-month adherence to guideline-directed medical therapies following percutaneous coronary intervention (PCI).

## Introduction

A significant proportion of individuals with coronary artery disease have or develop comorbid depression.^1^ The presence of depression is associated with a 4-fold increased risk of cardiovascular morbidity and mortality compared with its absence.^2^ One contributor toward this may be the association between depression and medication adherence.^3^ Individuals with cardiovascular disease and comorbid depression are significantly less likely to adhere to pharmacotherapy than those without depression.^4,5^ Studies examining the association between depression and medication adherence in patients with cardiovascular disease have been limited by absence of consideration of comorbid psychiatric conditions, pharmacologic treatment for depression, and social factors (eg, educational attainment, income, and insurance type) that may affect medication adherence.^6,7,8,9^

Adherence to guideline-directed medical therapies (GDMT), including antiplatelet agents, β-blockers, angiotensin-converting enzyme (ACE) inhibitors, angiotensin receptor blockers (ARBs), and statins, is critical for secondary prevention of cardiovascular disease.^10^ Importantly, identifying the association between depression and GDMT adherence may focus interventions to improve such prevention. To elucidate this association, we conducted a retrospective analysis of patients who underwent percutaneous coronary intervention (PCI) using a geographically and socially diverse Medicare and commercial health claims database. Our objective was to evaluate the association between depression and GDMT adherence accounting for multiple, potentially confounding clinical and social factors. We hypothesized that individuals with depression would have lower adherence to all classes of essential medications used for secondary cardiovascular disease prevention than those without depression.

## Methods

We used Optum Clinformatics DataMart, a US database with administrative claims data for more than 60 million individuals covered by Medicare and/or commercial insurance. Optum’s claims data originate from the inpatient, outpatient, and emergency department settings. The medical claims include *International Classification of Diseases, Ninth Revision, Clinical Modification* (*ICD-9-CM*) and *Tenth Revision, Clinical Modification* (*ICD-10-CM*) codes; site of service codes; Healthcare Common Procedure Coding System procedure codes; and *Current Procedural Terminology* version 4 procedure codes. Optum collects pharmacy and laboratory claims to ascertain data on medication utilization and laboratory values. The University of Pittsburgh institutional review board deemed this study exempt from human participant review and the requirement for informed consent given the deidentified nature of the data. We followed the Strengthening the Reporting of Observational Studies in Epidemiology (STROBE) reporting guideline.^11^

We included individuals who underwent PCI between January 1, 2014, to December 31, 2019. We used *Current Procedural Terminology* version 4 procedure codes (eTable 1 in the Supplement) to define PCI. For inclusion, individuals were required to have 12 months of continuous enrollment prior to PCI for adequate capture of comorbid medical and psychiatric conditions. Individuals were required to have 12 months of continuous enrollment after PCI as well to provide adequate time for the assessment of medication adherence. Individuals were excluded if they were discharged following PCI to long-term care or a skilled nursing facility, they did not have at least 12 months continuous enrollment either prior to or following the PCI, they were younger than 18 years, or they had missing sex information.

We identified a diagnosis of depression as occurring during the 12 months of enrollment prior to PCI or within 6 months following PCI. We defined depression according to the algorithm articulated by the Centers for Medicare & Medicaid Services Chronic Conditions Data Warehouse,^12^ which requires either 1 inpatient or 2 outpatient claims with *ICD* diagnosis codes for depression. Antidepressant prescription was not required for defining depression. *ICD-9-CM* and *ICD-10-CM* diagnostic codes for depression and its subtypes are provided in eTable 1 in the Supplement. We implemented both versions of *ICD* coding to capture the greatest number of depression diagnoses possible using administrative claims. During the change from *ICD-9-CM* to *ICD-10-CM* diagnostic coding, the prevalence of depression was steadily increasing.^13^ However, between 2014 and 2019, the rate of increase was negligible.

We assessed medication adherence with proportion of days covered (PDC), calculated as the ratio of the number of days a medication is available and the number of follow-up observation days.^14^ PDC is a standardized measure for claims-based adherence ascertainment and is validated by the Pharmacy Quality Alliance and Centers for Medicare & Medicaid Services.^15^ Medication discontinuation, incorporated into the calculation of PDC, is defined by a period of 60 or more days without available medication. A PDC of 80% or higher has been consistently used to consider an individual adherent to medication, while PDC of 90% or higher is considered optimal adherence.^16^ Given these values, we categorized PDC as adequate (≥80% to <90%) or optimal (≥90%).

We used outpatient pharmacy claims to identify medications. Claims data provided the number of days supplied, the fill date, and medication generic name. We included medications that represent GDMT for secondary coronary disease prevention as recommended by professional society guidelines^10^ (eTable 2 in the Supplement). We categorized these medications by class as antiplatelets (excluding aspirin), β-blockers, renin-angiotensin-aldosterone system (RAAS) inhibitors (combining ACEs and ARBs, as these agents are rarely used simultaneously), and statins. We identified medication changes within each class of GDMT agents to calculate PDC for each GDMT class, rather than individual medications. We also assessed antidepressant (selective serotonin reuptake inhibitors and serotonin-norepinephrine reuptake inhibitors) claims for all individuals.

Age, sex, and race or ethnicity were provided directly from the claims data. Optum categorizes race or ethnicity as Asian, Black, Hispanic, White, or unknown. Cardiac and noncardiac comorbidities listed in the Elixhauser Comorbidity Index^17^ were defined by *ICD-9-CM* and *ICD-10-CM* diagnosis codes and ascertained from inpatient and outpatient medical claims prior to PCI. We similarly identified comorbid psychiatric diagnoses, specifically bipolar disorder, schizophrenia, personality disorder, anxiety disorder, and posttraumatic stress disorder.

Optum uses US census data at the level of 9-digit zip code to classify level of education. Educational attainment is categorized as less than 12th grade education, high school diploma, some college education, bachelor’s degree or higher, or unknown. Optum obtains estimates of household income likewise using the 9-digit zip code from a consumer marketing database, as previously reported.^18,19^ Annual household income is categorized by Optum as less than $40 000, $40 000 to less than $50 000, $50 000 to less than $60 000, $60 000 to less than $75 000, $75 000 to less than $100 000, $100 000 or greater, or unknown. As such, these determinations are obtained at essentially the neighborhood level, rather than the individual level. Insurance type is categorized as Medicare or commercial. Number of GDMT agents was defined as the classes of medications used during the follow-up period. We further adjusted for GDMT regimen modification, ie, a change within class of agents, with the rationale that such modification may affect medication adherence.

### Statistical Analysis

We first summarized the distributions of baseline characteristics for all individuals, then compared them by depression status. We then calculated the PDC for each medication among all individuals. Using multivariable-adjusted logistic regression models, we calculated odds of adequate or optimal adherence in individuals with depression compared with those without depression.

We used 3 models for multivariable adjustment to address potential confounders. Model 1 adjusted for age, sex, and race and ethnicity; model 2 adjusted for variables included in model 1 as well as the number of Elixhauser Comorbidity Index conditions and comorbid psychiatric diagnoses (schizophrenia, personality disorder, anxiety disorder, and posttraumatic stress disorder). Model 3 adjusted for the variables included in model 2 as well as educational attainment, annual household income, insurance type, total number of medications, and switching medication during follow-up. Because no independent variables, covariates, or outcomes were missing, individuals with unknown race or ethnicity, education, and income were included in all analyses.

We conducted the following sensitivity analyses: (1) exclusion of individuals with existing psychiatric diagnoses (anxiety disorder, bipolar disorder, personality disorder, posttraumatic stress disorder, or schizophrenia) prior to PCI; (2) defining depression by contemporaneous use of a selective serotonin reuptake inhibitor, serotonin and norepinephrine reuptake inhibitor, or bupropion in addition to diagnostic codes; and (3) exclusion of all individuals receiving GDMT medications prior to PCI. *P* < .05 was used for statistical significance, and all tests were 2-tailed. We used SAS, version 9.4 (SAS Institute) for statistical analyses. Data analysis was conducted between February and August 2022.

## Results

A total of 247 505 individuals were identified as having PCI between 2014 and 2019. As shown in the eFigure in the Supplement, following application of inclusion and exclusion criteria, 124 443 individuals who underwent PCI (mean [SD] age, 69.3 [10.6] years; 41 430 [33.3%] female sex; 3694 [3.0%] Asian, 12 611 [10.1%] Black, and 12 337 [9.9%] Hispanic individuals) were included in the sample. Among important characteristics described in Table 1, the median (IQR) number of Elixhauser comorbidities was 6 (3-9). More than 72% of patients had Medicare (89 873 [72.2%]), and the median (IQR) number of GDMT classes of medication used was 3 (3-4).

**Table 1.** Characteristics of Percutaneous Coronary Intervention Cohort

Characteristic	Individuals, No. (%)		
	All (N = 124 443)	Without depression (n = 103 732)	With depression (n = 20 711)
Age, mean (SD), y	69.3 (10.6)	69.4 (10.7)	69.1 (10.2)
Sex			
Female	41 430 (33.3)	30 917 (29.8)	10 513 (50.8)
Male	83 013 (66.7)	72 815 (70.2)	10 198 (49.2)
Race			
Asian	3694 (3.0)	3424 (3.3)	270 (1.3)
Black	12 611 (10.1)	10 521 (10.1)	2090 (10.1)
Hispanic	12 337 (9.9)	10 368 (10.0)	1969 (9.5)
White	91 272 (73.3)	75 651 (72.9)	15 621 (75.4)
Unknown	4529 (3.6)	3768 (3.6)	761 (3.7)
No. of medical comorbidities, median (IQR)	6 (3-9)	5 (3-8)	9 (6-12)
Psychiatric comorbidities			
Bipolar disorder	4258 (3.4)	1834 (1.8)	2424 (11.7)
Schizophrenia	594 (0.5)	263 (0.3)	331 (1.6)
Personality disorder	2821 (2.3)	997 (1.0)	1824 (8.8)
Anxiety disorder	31 574 (25.4)	19 351 (18.7)	12 223 (59.0)
PTSD	1177 (1.0)	469 (0.5)	708 (3.4)
Educational attainment			
<12th grade	563 (0.5)	476 (0.5)	87 (0.4)
High school diploma	37 792 (30.4)	30 836 (29.7)	6956 (33.6)
<Bachelor’s degree	67 247 (54.0)	56 190 (54.2)	11 057 (53.4)
≥Bachelor’s degree	16 436 (13.2)	14 269 (13.8)	2167 (10.5)
Unknown	2405 (1.9)	1961 (1.9)	444 (2.1)
Household income, $			
<40 000	36 205 (29.1)	28 451 (27.4)	7754 (37.4)
40 000 to <50 000	11 283 (9.1)	9314 (9.0)	1969 (9.5)
50 000 to <60 000	11 563 (9.3)	9697 (9.4)	1866 (9.0)
60 000 to <75 000	14 456 (11.6)	12 231 (11.8)	2225 (10.7)
75 000 to <100 000	18 894 (15.2)	16 230 (15.7)	2664 (12.9)
≥100 000	25 230 (20.3)	22 370 (21.6)	2860 (13.8)
Unknown	6812 (5.5)	5439 (5.2)	1373 (6.6)
Medicare insurance	89 873 (72.2)	72 829 (70.2)	17 044 (82.3)
GDMT medication classes, No.			
1	729 (0.6)	620 (0.6)	109 (0.5)
2	10 868 (8.7)	9167 (8.8)	1701 (8.2)
3	64 414 (51.8)	53 589 (51.7)	10 825 (52.3)
4	46 374 (37.3)	38 613 (37.2)	7761 (37.5)
5	2058 (1.7)	1743 (1.7)	315 (1.5)
GDMT classes claims			
Antiplatelets	12 1912 (98.0)	101 647 (98.0)	20 265 (97.8)
β-blockers	111 851 (89.9)	92 902 (89.6)	18 949 (91.5)
ACE inhibitors	10 964 (8.8)	9335 (9.0)	1629 (7.9)
ARBs	47 400 (38.1)	39 468 (38.0)	7932 (38.3)
Statins	119 366 (95.9)	99 536 (96.0)	19 830 (95.7)
Prescribed antidepressants	26 182 (21.0)	12 121 (11.7)	14 061 (67.9)

Abbreviations: ACE, angiotensin-converting enzyme; ARBs, aldosterone receptor blockers; GDMT, guideline-directed medical therapy; PTSD, posttraumatic stress disorder.

There were 20 711 individuals (16.6%) identified as having depression (mean [SD] age, 69.1 [10.2] years, 10 513 [50.8%] female sex, 270 [1.3%] Asian, 2090 [10.1%] Black, and 1969 [9.5%] Hispanic individuals). The median (IQR) number of comorbidities in those with depression was 9 (6-12), compared with a median (IQR) of 5 (3-8) in those without depression. A higher proportion of individuals with depression had psychiatric comorbidities compared with those without, with anxiety disorders being the most prevalent diagnosis.

**Figure 1.**
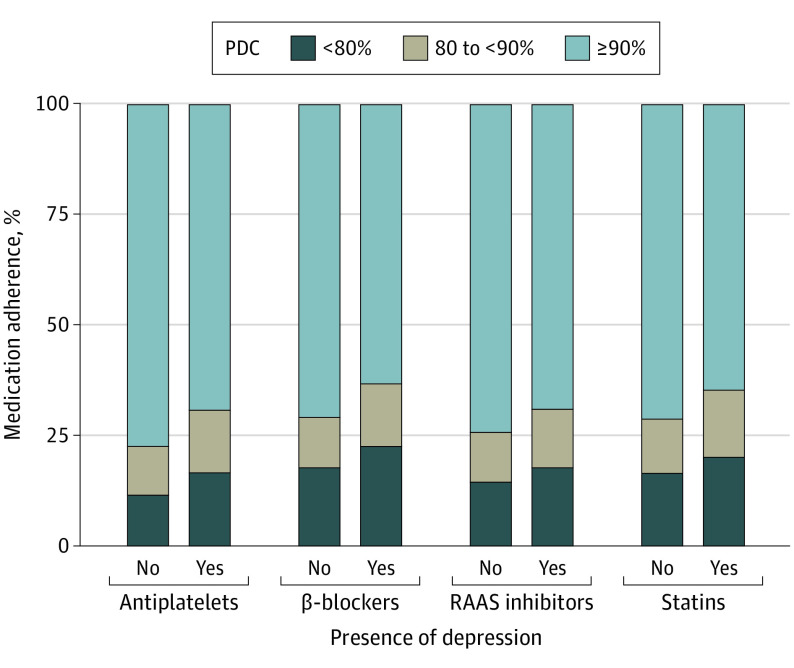
Percentage of Individuals With Limited, Adequate, and Optimal Adherence to Essential Medications Following Percutaneous Coronary Intervention Defined by Proportion of Days Covered (PDC) Bar graph of the percentage of individuals with limited (PDC <80%), adequate (PDC ≥80% to <90%), and optimal (PDC ≥90%) adherence to each essential class of medication. Individuals are categorized based on depression status. Renin-angiotensin-aldosterone system (RAAS) inhibitors include angiotensin-converting enzyme inhibitors and angiotensin receptor blockers.

**Figure 2.**
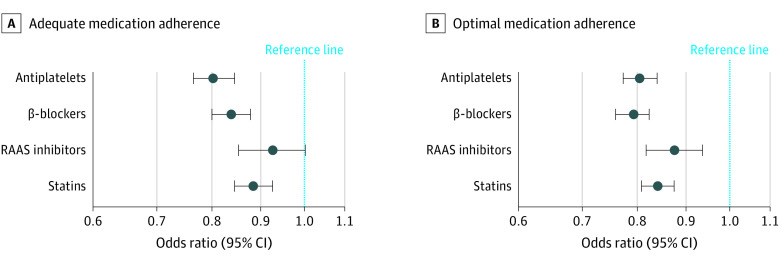
Odds of Adherence to Essential Medications Following Percutaneous Coronary Intervention by Depression Status Adjusted odds ratios with 95% CIs for adequate medication adherence, defined as at least 80% to less than 90% of proportion of days covered (A), and optimal medication adherence, defined as at least 90% of proportion of days covered (B), for each class of medication following percutaneous coronary intervention, comparing individuals with depression with those without. Odds ratios of medication adherence were calculated using multivariable-adjusted logistic regression model 3, as described in the Statistical Analysis section. Renin-angiotensin-aldosterone system (RAAS) inhibitors include angiotensin-converting enzyme inhibitors and angiotensin receptor blockers.

More individuals with depression had limited adherence to each essential component of GDMT compared with those without. Table 2 presents the proportion of individuals with and without depression and the proportion of adequate and optimal adherence by medication class. Figure 1 graphically summarizes the distribution of adherence by medication class and depression status. In fully adjusted logistic regression models, individuals with depression were significantly less likely to obtain adequate 12-month adherence to antiplatelets (odds ratio [OR], 0.80; 95% CI, 0.77-0.85), β-blockers (OR, 0.84; 95% CI, 0.80-0.88), and statins (OR, 0.88; 95% CI, 0.85-0.93) than those without depression (Table 3). Depression diagnosis was not significantly associated with adequate adherence to RAAS inhibitors (OR, 0.93; 95% CI, 0.85-1.00). Those with depression were similarly significantly less likely to obtain optimal 12-month adherence to antiplatelets (OR, 0.81; 95% CI, 0.77-0.84), β-blockers (OR, 0.79; 95% CI, 0.76-0.82), RAAS inhibitors (OR, 0.87; 95% CI, 0.82-0.94), and statins (OR, 0.84; 95% CI, 0.81-0.87) than those without after adjustment. Figure 2 graphically summarizes the association between depression and adherence by GDMT agent class.

**Table 2.** Proportion of Individuals With Adequate and Optimal Adherence to Guideline-Directed Medical Therapies Following Percutaneous Coronary Intervention

Medication class	Individuals, % (95% CI)		*P* value
	Without depression	With depression	
**Adequate adherence (PDC ≥80% to <90%)**			
Antiplatelets	11.1 (10.9-11.3)	14.2 (13.8-14.7)	<.001
β-blockers	11.4 (11.2-11.6)	14.2 (13.7-14.7)	<.001
RAAS inhibitors	13.2 (12.9-13.5)	16.4 (15.6-17.2)	<.001
Statins	12.3 (12.1-12.5)	15.2 (14.7-15.7)	<.001
**Optimal adherence (PDC ≥90%)**			
Antiplatelets	77.6 (77.4-77.9)	69.4 (68.8-70.0)	<.001
β-blockers	71.1 (70.8-71.3)	63.4 (62.7-64.1)	<.001
RAAS inhibitors	86.8 (86.5-87.1)	83.6 (82.8-84.4)	<.001
Statins	71.4 (71.1-71.7)	64.8 (64.2-65.5)	<.001

Abbreviations: PDC, proportion of days covered; RAAS, renin-angiotensin-aldosterone system.

**Table 3.** Odds of Adherence to Guideline-Directed Medical Therapies Following Percutaneous Coronary Intervention in Individuals With Depression Compared With Those Without Depression

Medication class	Model 1^a^		Model 2^b^		Model 3^c^	
	OR (95% CI)	*P* value	OR (95% CI)	*P* value	OR (95% CI)	*P* value
**Adequate adherence (PDC ≥80% to <90%)**						
Antiplatelets	0.65 (0.63-0.68)	<.001	0.79 (0.76-0.83)	<.001	0.80 (0.77-0.85)	<.001
β-blockers	0.74 (0.71-0.77)	<.001	0.84 (0.81-0.88)	<.001	0.84 (0.80-0.88)	<.001
RAAS inhibitors	0.80 (0.76-0.85)	<.001	0.92 (0.87-0.99)	.02	0.93 (0.85-1.00)	.06
Statins	0.79 (0.76-0.83)	<.001	0.90 (0.86-0.94)	<.001	0.88 (0.85-0.93)	<.001
**Optimal adherence (PDC ≥90%)**						
Antiplatelets	0.66 (0.63-0.68)	<.001	0.79 (0.76-0.82)	<.001	0.81 (0.77- 0.84)	<.001
β-blockers	0.71 (0.68-0.73)	<.001	0.81 (0.78-0.84)	<.001	0.79 (0.76-0.82)	<.001
RAAS inhibitors	0.78 (0.74-0.82)	<.001	0.89 (0.85-0.95)	<.001	0.87 (0.82-0.94)	<.001
Statins	0.75 (0.73-0.78)	<.001	0.85 (0.82-0.88)	<.001	0.84 (0.81-0.87)	<.001

Abbreviations: OR, odds ratio; PDC, proportion of days covered; RAAS, renin-angiotensin-aldosterone system.

^a^ Model 1 was adjusted for age, sex, and race and ethnicity.

^b^ Model 2 was adjusted for age, sex, race and ethnicity, number of medical comorbidities, and comorbid psychiatric conditions.

^c^ Model 3 was adjusted for age, sex, race and ethnicity, number of medical comorbidities, comorbid psychiatric conditions, educational attainment, income level, insurance type, number of medications used, and switching medication during follow-up.

Results from sensitivity analyses are presented in eTables 3 and 4 in the Supplement. The exclusion of those with psychiatric diagnosis, those requiring use of common antidepressant medications in addition to diagnostic coding, and those with GDMT agent use prior to PCI yielded estimates similar to the multivariable-adjusted results summarized in Table 3 with respect to adequate and optimal adherence to antiplatelets, β-blockers, and statins. We did not observe an association between depression and adherence to RAAS inhibitors, likely given the heterogenous indications for this class of medications.

## Discussion

We examined the association between depression and 12-month adherence to essential GDMT agents in a large-sized cohort of individuals following PCI. Those identified as having depression were significantly less likely to achieve either adequate or optimal levels of 12-month adherence to each class of GDMT agent. Our most noteworthy finding was the difference in adherence to antiplatelet agents, with depression being associated with an approximately 20% lower likelihood of adequate (OR, 0.80; 95% CI, 0.77-0.85) or optimal (OR, 0.81; 95% CI, 0.77-0.84) adherence compared with its absence. This finding is of particular concern given enrollment in our cohort was defined by having had a PCI. Our results demonstrate crucial opportunities for interventions to augment secondary cardiovascular disease prevention in all individuals, especially those with depression.

Medication adherence is an expected but highly complex component of primary and secondary cardiovascular disease prevention. The World Health Organization for medication adherence framework recognizes 5 main determinants: socioeconomic resources, the health system, diagnosis and disease, medication selection, and the individual patient.^20^ The overlap of these domains with each other has further capacity to exacerbate challenges to medication adherence. Socioeconomic factors are an evident barrier to medication adherence, particularly as large segments of the US population face the increasing financial strain from routine expenses such as housing, food, and childcare. The net result is a limited affordability of out-of-pocket medication costs, alongside additional social barriers, such as absence of transportation or residing in a pharmacy desert. Furthermore, secondary cardiovascular prevention requires an array of therapies from multiple medication classes; the resulting polypharmacy and complexity of dosing regimen may contribute to diminished adherence. Selection of nongeneric or costlier medications may in turn also yield higher copays. Finally, health literacy presents an additional, complex barrier that interacts with multiple social determinants to affect adherence to cardiovascular therapies.^21^

Medication adherence is a pillar of secondary prevention of cardiovascular disease. Long-term adherence to antiplatelet agents, frequently a minimum of 12 months, is essential to maintain stent patency; individuals with limited medication adherence following coronary events are at increased risk for recurrent infarction, heart failure, and death.^22^ Nonadherence and its clinical consequences contribute to significantly increased health care costs, rehospitalizations, and overuse of medical services.^23^ Nevertheless, the net effect of myriad factors at the individual and health care delivery system level is that diminished adherence to GDMT agents is highly prevalent.^24^

Depression is well-recognized as augmenting primary and secondary cardiovascular risk, as well summarized elsewhere.^25,26^ Individuals with depression have up to 3-fold increased risk of morbidity and mortality after myocardial infarction compared with those without depression.^27^ The mechanisms are multifactorial given the biological and social processes associated with depression. Depression is associated with autonomic dysfunction, hormonal dysregulation, hypercoagulability, and endothelial inflammation, all of which may augment cardiovascular risk.^28^ Individuals with depression have increased likelihood of an array of behavioral changes.^29^ Behavioral factors, such as socialization, physical activity, dietary selection, and smoking, were not accounted for in our study. However, we recognize that behavioral risk factors for cardiovascular disease are not reliably characterized by an administrative database.

Our work extends the prior literature on depression and cardiovascular disease by reporting the contribution of depression to 12-month adherence to GDMT following PCI. These results indicate the critical importance of strategies to address depression as part of secondary cardiovascular disease prevention. The American Heart Association has advised depression screening for all patients with coronary disease given the adverse contribution of depression to cardiac prognosis.^30^ However, depression commonly goes unrecognized and undertreated in clinical settings.^31^ Either due to resistance from patients to report their depressive symptoms or poor screening practices, our findings of depression negatively affecting medication adherence could be underestimated. We advocate for multidisciplinary interventions to ensure frequent follow-ups, prescription reminders, and counseling to target challenges to medical adherence. As we relied on administrative coding to identify depression, we expect that depression is far more prevalent than identified in our study. The identification and treatment of depression in patients with coronary disease may also improve health-related quality of life, mood, and functional status,^32^ which in turn may reduce behavioral challenges to medication adherence.

Our study has several strengths. We conducted our analysis in a large data set, thereby increasing its generalizability. We included 124 443 individuals’ Medicare and commercial insurance health care claims from diverse geographic regions of the United States. A further strength is our multivariable adjustment for comorbid psychiatric conditions and social factors (educational attainment and annual household income) which may confound the association between depression and GDMT medication adherence. We consider inclusion of these covariates as distinguishing our study from prior studies examining depression and medication adherence.

### Limitations

This study has limitations. First, only individuals with insurance were included in the analysis, such that results may not be generalizable to those without insurance. Second, there is potential for misclassification of depression status due to our reliance on claims data and *ICD* coding. We expect that our use of a claims-based diagnosis may result in underdiagnosis of depression in addition to precluding an assessment of its severity. Third, adherence was determined from records of prescription fills and consequently does not reflect individuals’ daily use of medications. More direct determination of adherence (such as directly observed therapy or pill counts) would not be practical with administrative data, and PDC is used extensively to assess adherence in claims data. Fourth, we recognize potential for residual confounding from unmeasured variables, such as multiple social and behavioral factors that may be associated with depression and adherence. Additionally, we were not able to ascertain reasons for medication discontinuation, such as adverse effects. However, we expect the resulting misclassification of PDC would be nondifferential and therefore biased toward the null.

## Conclusions

This cohort study of health care claims found an association between depression and likelihood of adequate or optimal 12-month adherence to essential GDMT agents following PCI. Further studies are needed to determine whether treatment of depression may improve medication adherence as well as how such treatment improves secondary prevention of cardiovascular disease.

## Notes

Supplement. **eTable 1.** Diagnosis and Procedure Codes Used for Comorbidity Ascertainment and Cohort Selection **eTable 2.** Medications and Respective Classes Used in Analysis **eTable 3.** Sensitivity Analyses for Adherence to Guideline-Directed Medical Therapies Following Coronary Intervention in Individuals With Depression Compared With Those Without Depression **eTable 4.** Sensitivity Analysis for Adherence (Adequate and Optimal) to Guideline-Directed Medical Therapies Following Coronary Intervention in Individuals With Depression Compared With Those Without Depression **eFigure.** Cohort Selection Flow Chart
