# Supplementary material for: Assessment of Depression and Adherence to Guideline-Directed Medical Therapies Following Percutaneous Coronary Intervention
Source: JAMA Netw Open. 2022 Dec 12;5(12):e2246317. doi: 10.1001/jamanetworkopen.2022.46317 (PMC9856464; doi:10.1001/jamanetworkopen.2022.46317)
Supplement: Supplement. — eTable 1. Diagnosis and Procedure Codes Used for Comorbidity Ascertainment and Cohort Selection eTable 2. Medications and Respective Classes Used in Analysis eTable 3. Sensitivity Analyses for Adherence to Guideline-Directed Medical Therapies Following Coronary Intervention in Individuals With Depression Compared With Those Without Depression eTable 4. Sensitivity Analysis for Adherence (Adequate and Optimal) to Guideline-Directed Medical Therapies Following Coronary Intervention in Individuals With Depression Compared With Those Without Depression eFigure. Cohort Selection Flow Chart [file jamanetwopen-e2246317-s001.pdf]

Supplemental Online Content

Lapa ME, Swabe GM, Rollman BL, Muldoon MF, Thurston RC, Magnani JW. Assessment of depression and adherence to guideline-directed medical therapies following percutaneous coronary intervention. *JAMA Netw Open.* 2022;5(12):e2246317. doi:10.1001/jamanetworkopen.2022.46317

eTable 1. Diagnosis and Procedure Codes Used for Comorbidity Ascertainment and Cohort Selection

eTable 2. Medications and Respective Classes Used in Analysis

eTable 3. Sensitivity Analyses for Adherence to Guideline-Directed Medical Therapies Following Coronary Intervention in Individuals With Depression Compared With Those Without Depression

eTable 4. Sensitivity Analysis for Adherence (Adequate and Optimal) to Guideline-Directed Medical Therapies Following Coronary Intervention in Individuals With Depression Compared With Those Without Depression

eFigure. Cohort Selection Flow Chart

This supplemental material has been provided by the authors to give readers additional information about their work.

© 2022 Lapa ME et al. *JAMA Network Open.*

eTable 1. Diagnosis and Procedure Codes Used for Comorbidity Ascertainment and Cohort Selection.	
**Condition**	**Diagnosis Codes (ICD-9-CM and ICD-10-CM)**
CHF	I09.81, I11.0, I13.0, I13.2, I50.1, I50.20, I50.21, I50.22, I50.23, I50.30, I50.31, I50.32, I50.33, I50.40, I50.41, I50.42, I50.43, I50.810, I50.811, I50.812, I50.813, I50.814, I50.82, I50.83, I50.84, I50.89, I50.9, I51.81, I97.130, I97.131, O29.121, O29.122, O29.123, O29.129, R57.0, Z95.811, Z95.812, 398.91, 402.01, 402.11, 402.91, 404.01, 404.03, 404.11, 404.13, 404.91, 404.93, 428.0, 428.1, 428.2, 428.3, 428.4, 428.5, 428.6, 428.7, 428.8, 428.9
Valvular disease	A18.84, A32.82, A39.51, A52.03, B33.21, B37.6, I01.1, I01.8, I01.9, I02.0, I05.0, I05.1, I05.2, I05.8, I05.9, I06.0, I06.1, I06.2, I06.8, I06.9, I07.0, I07.1, I07.2, I07.8, I07.9, I08.0, I08.1, I08.2, I08.3, I08.8, I08.9, I09.1, I09.89, I33.0, I33.9, I34.0, I34.1, I34.2, I34.8, I34.9, I35.0, I35.1, I35.2, I35.8, I35.9, I36.0, I36.1, I36.2, I36.8, I36.9, I37.0, I37.1, I37.2, I37.8, I37.9, I38.X, I39.X, M32.11, Q22.0, Q22.1, Q22.2, Q22.3, Q22.4, Q22.5, Q22.6, Q22.8, Q22.9, Q23.0, Q23.1, Q23.2, Q23.3, Q23.4, Q23.8, Q23.9, T82.01XA, T82.01XD, T82.01XS, T82.02XA, T82.02XD, T82.02XS, T82.03XA, T82.03XD, T82.03XS, T82.09XA, T82.09XD, T82.09XS, T82.221A, T82.221D, T82.221S, T82.222A, T82.222D, T82.222S, T82.223A, T82.223D, T82.223S, T82.228A, T82.228D, T82.228S, T82.6XXA, T82.6XXD, T82.6XXS, Z95.2, Z95.3, Z95.4, 932.0, 932.1, 932.2, 932.3, 932.4, 394.0, 394.1, 394.2, 394.3, 394.4, 394.5, 394.6, 394.7, 394.8, 394.9, 395.0, 395.1, 395.2, 395.3, 395.4, 395.5, 395.6, 395.7, 395.8, 395.9, 396.0, 396.1, 396.2, 396.3, 396.4, 396.5, 396.6, 396.7, 396.8, 396.9, 397.0, 397.1, 397.9, 424.0, 424.01, 424.02, 424.03, 424.04, 424.05, 424.06, 424.07, 424.08, 424.09, 424.10, 424.11, 424.12, 424.13, 424.14, 424.15, 424.16, 424.17, 424.18, 424.19, 424.20, 424.21, 424.22, 424.23, 424.24, 424.25, 424.26, 424.27, 424.28, 424.29, 424.30, 424.31, 424.32, 424.33, 424.34, 424.35, 424.36, 424.37, 424.38, 424.39, 424.40, 424.41, 424.42, 424.43, 424.44, 424.45, 424.46, 424.47, 424.48, 424.49, 424.50, 424.51, 424.52, 424.53, 424.54, 424.55, 424.56, 424.57, 424.58, 424.59, 424.60, 424.61, 424.62, 424.63, 424.64, 424.65, 424.66, 424.67, 424.68, 424.69, 424.70, 424.71, 424.72, 424.73, 424.74, 424.75, 424.76, 424.77, 424.78, 424.79, 424.80, 424.81, 424.82, 424.83, 424.84, 424.85, 424.86, 424.87, 424.88, 424.89, 424.90, 424.91, 424.92, 424.93, 424.94, 424.95, 424.96, 424.97, 424.98, 424.99, 746.3, 746.4, 746.5, 746.6, V42.2, V43.3
Pulmonary circulation disorders	I27.0, I27.1, I27.2, I27.20, I27.21, I27.22, I27.23, I27.24, I27.29, I27.81, I27.82, I27.83, I27.89, I27.9, I28.0, I28.1, I28.8, I28.9, 415.11, 415.12, 415.13, 415.14, 415.15, 415.16, 415.17, 415.18, 415.19, 416.0, 416.1, 416.2, 416.3, 416.4, 416.5, 416.6, 416.7, 416.8, 416.9, 417.9

© 2022 Lapa ME et al. *JAMA Network Open.*

Peripheral vascular disorders	A52.00, A52.01, A52.02, A52.09, I70.0, I70.1, I70.201, I70.202, I70.203, I70.208, I70.209, I70.211, I70.212, I70.213, I70.218, I70.219, I70.221, I70.222, I70.223, I70.228, I70.229, I70.231, I70.232, I70.233, I70.234, I70.235, I70.238, I70.239, I70.241, I70.242, I70.243, I70.244, I70.245, I70.248, I70.249, I70.25, I70.261, I70.262, I70.263, I70.268, I70.269, I70.291, I70.292, I70.293, I70.298, I70.299, I70.301, I70.302, I70.303, I70.308, I70.309, I70.311, I70.312, I70.313, I70.318, I70.319, I70.321, I70.322, I70.323, I70.328, I70.329, I70.331, I70.332, I70.333, I70.334, I70.335, I70.338, I70.339, I70.341, I70.342, I70.343, I70.344, I70.345, I70.348, I70.349, I70.35, I70.361, I70.362, I70.363, I70.368, I70.369, I70.391, I70.392, I70.393, I70.398, I70.399, I70.401, I70.402, I70.403, I70.408, I70.409, I70.411, I70.412, I70.413, I70.418, I70.419, I70.421, I70.422, I70.423, I70.428, I70.429, I70.431, I70.432, I70.433, I70.434, I70.435, I70.438, I70.439, I70.441, I70.442, I70.443, I70.444, I70.445, I70.448, I70.449, I70.45, I70.461, I70.462, I70.463, I70.468, I70.469, I70.491, I70.492, I70.493, I70.498, I70.499, I70.501, I70.502, I70.503, I70.508, I70.509, I70.511, I70.512, I70.513, I70.518, I70.519, I70.521, I70.522, I70.523, I70.528, I70.529, I70.531, I70.532, I70.533, I70.534, I70.535, I70.538, I70.539, I70.541, I70.542, I70.543, I70.544, I70.545, I70.548, I70.549, I70.55, I70.561, I70.562, I70.563, I70.568, I70.569, I70.591, I70.592, I70.593, I70.598, I70.599, I70.601, I70.602, I70.603, I70.608, I70.609, I70.611, I70.612, I70.613, I70.618, I70.619, I70.621, I70.622, I70.623, I70.628, I70.629, I70.631, I70.632, I70.633, I70.634, I70.635, I70.638, I70.639, I70.641, I70.642, I70.643, I70.644, I70.645, I70.648, I70.649, I70.65, I70.661, I70.662, I70.663, I70.668, I70.669, I70.691, I70.692, I70.693, I70.698, I70.699, I70.701, I70.702, I70.703, I70.708, I70.709, I70.711, I70.712, I70.713, I70.718, I70.719, I70.721, I70.722, I70.723, I70.728, I70.729, I70.731, I70.732, I70.733, I70.734, I70.735, I70.738, I70.739, I70.741, I70.742, I70.743, I70.744, I70.745, I70.748, I70.749, I70.75, I70.761, I70.762, I70.763, I70.768, I70.769, I70.791, I70.792, I70.793, I70.798, I70.799, I70.8, I70.90, I70.91, I70.92, I71.00, I71.01, I71.02, I71.03, I71.1, I71.2, I71.3, I71.4, I71.5, I71.6, I71.8, I71.9, I72.0, I72.1, I72.2, I72.3, I72.4, I72.5, I72.6, I72.8, I72.9, I73.01, I73.1, I73.81, I73.89, I73.9, I74.01, I74.09, I74.10, I74.11, I74.19, I74.2, I74.3, I74.4, I74.5, I74.8, I74.9, I75.011, I75.012, I75.013, I75.019, I75.021, I75.022, I75.023, I75.029, I75.81, I75.89, I77.0, I77.1, I77.2, I77.3, I77.4, I77.5, I77.6, I77.70, I77.71, I77.72, I77.73, I77.74, I77.75, I77.76, I77.77, I77.79, I77.810, I77.811, I77.812, I77.819, I77.89, I77.9, I78.0, I78.1, I78.8, I78.9, I79.0, I79.1, I79.8, I99.8, I99.9, K31.811, K31.819, K55.1, K55.8, K55.9, Z95.820, Z95.828, 440.X, 440.1, 440.2, 440.3, 440.4, 440.5, 440.6, 440.7, 440.8, 440.9, 441.00, 441.01, 441.02, 441.03, 441.04, 441.05, 441.06, 441.07, 441.08, 441.09, 441.10, 441.11, 441.12, 441.13, 441.14, 441.15, 441.16, 441.17, 441.18, 441.19, 441.20, 441.21, 441.22, 441.23, 441.24, 441.25, 441.26, 441.27, 441.28, 441.29, 441.30, 441.31, 441.32, 441.33, 441.34, 441.35, 441.36, 441.37, 441.38, 441.39, 441.40, 441.41, 441.42, 441.43, 441.44, 441.45, 441.46, 441.47, 441.48, 441.49, 441.50, 441.51, 441.52, 441.53, 441.54, 441.55, 441.56, 441.57, 441.58, 441.59, 441.60, 441.61, 441.62, 441.63, 441.64, 441.65, 441.66, 441.67, 441.68, 441.69, 441.70, 441.71, 441.72, 441.73, 441.74, 441.75, 441.76, 441.77, 441.78, 441.79, 441.80, 441.81, 441.82, 441.83, 441.84, 441.85, 441.86, 441.87, 441.88, 441.89, 441.90, 442.0, 442.1, 442.2, 442.3, 442.4, 442.5, 442.6, 442.7, 442.8, 442.9, 443.1, 443.2, 443.3, 443.4, 443.5, 443.6, 443.7, 443.8, 443.9, 444.21, 444.22, 444.71, 449.X, 557.1, 557.9, V43.4

© 2022 Lapa ME et al. *JAMA Network Open.*

Hypertension (uncomplicated)	I10.X, I16.0, I16.9, O10.011, O10.012, O10.013, O10.019, O10.02, O10.03, 401.1, 401.9, 642.00, 642.01, 642.02, 642.03, 642.04
Hypertension (complicated)	H35.031, H35.032, H35.033, H35.039, I11.0, I11.9, I12.0, I12.9, I13.0, I13.10, I13.11, I13.2, I15.0, I15.1, I15.2, I15.8, I15.9, I16.1, I67.4, O10.111, O10.112, O10.113, O10.119, O10.12, O10.13, O10.211, O10.212, O10.213, O10.219, O10.22, O10.23, O10.311, O10.312, O10.313, O10.319, O10.32, O10.33, O10.411, O10.412, O10.413, O10.419, O10.42, O10.43, O10.911, O10.912, O10.913, O10.919, O10.92, O10.93, O11.1, O11.2, O11.3, O11.4, O11.5, O11.9, O16.1, O16.2, O16.3, O16.4, O16.5, O16.9, 401.0, 402.00, 402.01, 402.02, 402.03, 402.04, 402.05, 402.06, 402.07, 402.08, 402.09, 402.10, 402.11, 402.12, 402.13, 402.14, 402.15, 402.16, 402.17, 402.18, 402.19, 402.20, 402.21, 402.22, 402.23, 402.24, 402.25, 402.26, 402.27, 402.28, 402.29, 402.30, 402.31, 402.32, 402.33, 402.34, 402.35, 402.36, 402.37, 402.38, 402.39, 402.40, 402.41, 402.42, 402.43, 402.44, 402.45, 402.46, 402.47, 402.48, 402.49, 402.50, 402.51, 402.52, 402.53, 402.54, 402.55, 402.56, 402.57, 402.58, 402.59, 402.60, 402.61, 402.62, 402.63, 402.64, 402.65, 402.66, 402.67, 402.68, 402.69, 402.70, 402.71, 402.72, 402.73, 402.74, 402.75, 402.76, 402.77, 402.78, 402.81, 402.82, 402.83, 402.84, 402.85, 402.86, 402.87, 402.88, 402.89, 402.90, 402.91, 402.92, 402.93, 402.94, 402.95, 402.96, 402.97, 402.98, 402.99, 403.00, 403.01, 403.02, 403.03, 403.04, 403.05, 403.06, 403.07, 403.08, 403.09, 403.10, 403.11, 403.12, 403.13, 403.14, 403.15, 403.16, 403.17, 403.18, 403.19, 403.20, 403.21, 403.22, 403.23, 403.24, 403.25, 403.26, 403.27, 403.28, 403.29, 403.30, 403.31, 403.32, 403.33, 403.34, 403.35, 403.36, 403.37, 403.38, 403.39, 403.40, 403.41, 403.42, 403.43, 403.44, 403.45, 403.46, 403.47, 403.48, 403.49, 403.50, 403.51, 403.52, 403.53, 403.54, 403.55, 403.56, 403.57, 403.58, 403.59, 403.60, 403.61, 403.62, 403.63, 403.64, 403.65, 403.66, 403.67, 403.68, 403.69, 403.70, 403.71, 403.72, 403.73, 403.74, 403.75, 403.76, 403.77, 403.78, 403.79, 403.80, 403.81, 403.82, 403.83, 403.84, 403.85, 403.86, 403.87, 403.88, 403.89, 403.90, 403.91, 403.92, 403.93, 403.94, 403.95, 403.96, 403.97, 403.98, 403.99, 404.00, 404.01, 404.02, 404.03, 404.04, 404.05, 404.06, 404.07, 404.08, 404.09, 404.10, 404.11, 404.12, 404.13, 404.14, 404.15, 404.16, 404.17, 404.18, 404.19, 404.20, 404.21, 404.22, 404.23, 404.24, 404.25, 404.26, 404.27, 404.28, 404.29, 404.30, 404.31, 404.32, 404.33, 404.34, 404.35, 404.36, 404.37, 404.38, 404.39, 404.40, 404.41, 404.42, 404.43, 404.44, 404.45, 404.46, 404.47, 404.48, 404.49, 404.50, 404.51, 404.52, 404.53, 404.54, 404.55, 404.56, 404.57, 404.58, 404.59, 404.60, 404.61, 404.62, 404.63, 404.64, 404.65, 404.66, 404.67, 404.68, 404.69, 404.70, 404.71, 404.72, 404.73, 404.74, 404.75, 404.76, 404.77, 404.78, 404.79, 404.80, 404.81, 404.82, 404.83, 404.84, 404.85, 404.86, 404.87, 404.88, 404.89, 404.90, 404.91, 404.92, 404.93, 404.94, 404.95, 404.96, 404.97, 404.98, 404.99, 405.00, 405.01, 405.02, 405.03, 405.04, 405.05, 405.06, 405.07, 405.08, 405.09, 405.10, 405.11, 405.12, 405.13, 405.14, 405.15, 405.16, 405.17, 405.18, 405.19, 405.20, 405.21, 405.22, 405.23, 405.24, 405.25, 405.26, 405.27, 405.28, 405.29, 405.30, 405.31, 405.32, 405.33, 405.34, 405.35, 405.36, 405.37, 405.38, 405.39, 405.40, 405.41, 405.42, 405.43, 405.44, 405.45, 405.46, 405.47, 405.48, 405.49, 405.50, 405.51, 405.52, 405.53, 405.54, 405.55, 405.56, 405.57, 405.58, 405.59, 405.60, 405.61, 405.62, 405.63, 405.64, 405.65, 405.66, 405.67, 405.68, 405.69, 405.70, 405.71, 405.72, 405.73, 405.74, 405.75, 405.76, 405.77, 405.78, 405.79, 405.80, 405.81, 405.82, 405.83, 405.84, 405.85, 405.86, 405.87, 405.88, 405.89, 405.90, 405.91, 405.92, 405.93, 405.94, 405.95, 405.96, 405.97, 405.98, 405.99, 437.2, 642.10, 642.11, 642.12, 642.13, 642.14, 642.15, 642.16, 642.17, 642.18, 642.19, 642.20, 642.21, 642.22, 642.23, 642.24, 642.70, 642.71, 642.72, 642.73, 642.74, 642.75,

	642.76, 642.77, 642.78, 642.79, 642.80, 642.81, 642.82, 642.83, 642.84, 642.85, 642.86, 642.87, 642.88, 642.89, 642.90, 642.91, 642.92, 642.93, 642.94
Paralysis	G04.1, G80.0, G80.1, G80.2, G80.8, G80.9, G81.00, G81.01, G81.02, G81.03, G81.04, G81.10, G81.11, G81.12, G81.13, G81.14, G81.90, G81.91, G81.92, G81.93, G81.94, G82.20, G82.21, G82.22, G82.50, G82.51, G82.52, G82.53, G82.54, G83.0, G83.10, G83.11, G83.12, G83.13, G83.14, G83.20, G83.21, G83.22, G83.23, G83.24, G83.30, G83.31, G83.32, G83.33, G83.34, G83.4, G83.5, G83.81, G83.82, G83.83, G83.84, G83.89, G83.9, I69.031, I69.032, I69.033, I69.034, I69.039, I69.041, I69.042, I69.043, I69.044, I69.049, I69.051, I69.052, I69.053, I69.054, I69.059, I69.061, I69.062, I69.063, I69.064, I69.065, I69.069, I69.131, I69.132, I69.133, I69.134, I69.139, I69.141, I69.142, I69.143, I69.144, I69.149, I69.151, I69.152, I69.153, I69.154, I69.159, I69.161, I69.162, I69.163, I69.164, I69.165, I69.169, I69.231, I69.232, I69.233, I69.234, I69.239, I69.241, I69.242, I69.243, I69.244, I69.249, I69.251, I69.252, I69.253, I69.254, I69.259, I69.261, I69.262, I69.263, I69.264, I69.265, I69.269, I69.331, I69.332, I69.333, I69.334, I69.339, I69.341, I69.342, I69.343, I69.344, I69.349, I69.351, I69.352, I69.353, I69.354, I69.359, I69.361, I69.362, I69.363, I69.364, I69.365, I69.369, I69.831, I69.832, I69.833, I69.834, I69.839, I69.841, I69.842, I69.843, I69.844, I69.849, I69.851, I69.852, I69.853, I69.854, I69.859, I69.861, I69.862, I69.863, I69.864, I69.865, I69.869, I69.931, I69.932, I69.933, I69.934, I69.939, I69.941, I69.942, I69.943, I69.944, I69.949, I69.951, I69.952, I69.953, I69.954, I69.959, I69.961, I69.962, I69.963, I69.964, I69.965, I69.969, R53.2, 342.0, 342.1, 342.2, 342.3, 342.4, 342.5, 342.6, 342.7, 342.8, 342.9, 343.0, 343.1, 343.2, 343.3, 343.4, 343.5, 343.6, 343.7, 343.8, 343.9, 344.0, 344.1, 344.2, 344.3, 344.4, 344.5, 344.6, 344.7, 344.8, 344.9, 438.20, 438.21, 438.22, 438.23, 438.24, 438.25, 438.26, 438.27, 438.28, 438.29, 438.30, 438.31, 438.32, 438.33, 438.34, 438.35, 438.36, 438.37, 438.38, 438.39, 438.40, 438.41, 438.42, 438.43, 438.44, 438.45, 438.46, 438.47, 438.48, 438.49, 438.50, 438.51, 438.52, 438.53, 780.72
Neurological movement disorders	G08.X, G10.X, G11.0, G11.1, G11.10, G11.11, G11.19, G11.2, G11.3, G11.4, G11.8, G11.9, G12.0, G12.1, G12.20, G12.21, G12.22, G12.23, G12.24, G12.25, G12.29, G12.8, G12.9, G13.0, G13.1, G13.2, G13.8, G20.X, G21.0, G21.11, G21.19, G21.2, G21.3, G21.4, G21.8, G21.9, G23.0, G23.1, G23.2, G23.8, G23.9, G24.09, G24.1, G24.2, G24.8, G25.4, G25.5, G25.70, G25.71, G25.79, G25.81, G25.82, G25.83, G25.89, G25.9, G26.X, G32.0, G32.81, G32.89, G80.3
Seizures and epilepsy	G40.001, G40.009, G40.011, G40.019, G40.101, G40.109, G40.111, G40.119, G40.201, G40.209, G40.211, G40.219, G40.301, G40.309, G40.311, G40.319, G40.401, G40.409, G40.411, G40.419, G40.42, G40.501, G40.509, G40.801, G40.802, G40.803, G40.804, G40.811, G40.812, G40.813, G40.814, G40.821, G40.822, G40.823, G40.824, G40.833, G40.834, G40.89, G40.901, G40.909, G40.911, G40.919, G40.A01, G40.A09, G40.A11, G40.A19, G40.B01, G40.B09, G40.B11, G40.B19, R56.1, R56.9
Other neurological disorders	E75.00, E75.01, E75.02, E75.09, E75.10, E75.11, E75.19, E75.23, E75.25, E75.26, E75.29, E75.4, F05.X, F84.2, G35.X, G36.0, G36.8, G36.9, G37.0, G37.1, G37.2, G37.3, G37.4, G37.5, G37.8, G37.9, G47.411, G47.419, G47.421, G47.429, G89.0, G91.0, G91.1, G91.2, G91.3, G91.4, G91.8, G91.9, G93.0, G93.40, G93.41, G93.49, G93.5, G93.6, G93.7, G93.81, G93.82, G93.89, G93.9, G94.X, O99.350, O99.351, O99.352, O99.353, O99.354, O99.355, P91.60, P91.61, P91.62, P91.63, 330.1, 330.2, 330.3, 330.4, 330.5, 330.6, 330.7, 330.8, 330.9, 331.0, 331.1, 331.2, 331.3, 331.4, 331.5, 331.6, 331.7, 331.8, 331.9, 332.0,

© 2022 Lapa ME et al. *JAMA Network Open.*

	333.4, 333.5, 333.71, 333.72, 333.79, 333.85, 333.94, 334.0, 334.1, 334.2, 334.3, 334.4, 334.5, 334.6, 334.7, 334.8, 334.9, 335.0, 335.1, 335.2, 335.3, 335.4, 335.5, 335.6, 335.7, 335.8, 335.9, 338.0, 340.X, 341.1, 341.2, 341.3, 341.4, 341.5, 341.6, 341.7, 341.8, 341.9, 345.00, 345.01, 345.02, 345.03, 345.04, 345.05, 345.06, 345.07, 345.08, 345.09, 345.10, 345.11, 345.2, 345.3, 345.40, 345.41, 345.42, 345.43, 345.44, 345.45, 345.46, 345.47, 345.48, 345.49, 345.50, 345.51, 345.52, 345.53, 345.54, 345.55, 345.56, 345.57, 345.58, 345.59, 345.60, 345.61, 345.62, 345.63, 345.64, 345.65, 345.66, 345.67, 345.68, 345.69, 345.70, 345.71, 345.72, 345.73, 345.74, 345.75, 345.76, 345.77, 345.78, 345.79, 345.80, 345.81, 345.82, 345.83, 345.84, 345.85, 345.86, 345.87, 345.88, 345.89, 345.90, 345.91, 347.00, 347.01, 347.10, 347.11, 649.40, 649.41, 649.42, 649.43, 649.44, 768.7, 768.70, 768.71, 768.72, 780.3, 780.31, 780.32, 780.33, 780.39, 780.97, 784.3
Chronic pulmonary disease	J41.0, J41.1, J41.8, J42.X, J43.0, J43.1, J43.2, J43.8, J43.9, J44.0, J44.1, J44.9, J45.20, J45.21, J45.22, J45.30, J45.31, J45.32, J45.40, J45.41, J45.42, J45.50, J45.51, J45.52, J45.901, J45.902, J45.909, J45.990, J45.991, J45.998, J47.0, J47.1, J47.9, J60.X, J61.X, J62.0, J62.8, J63.0, J63.1, J63.2, J63.3, J63.4, J63.5, J63.6, J64.X, J65.X, J66.0, J66.1, J66.2, J66.8, J67.0, J67.1, J67.2, J67.3, J67.4, J67.5, J67.6, J67.7, J67.8, J67.9, J68.4, J70.1, J70.3, 490.X, 490.1, 490.2, 490.3, 490.4, 490.5, 490.6, 490.7, 490.8, 490.9, 491.0, 491.1, 491.2, 491.3, 491.4, 491.5, 491.6, 491.7, 491.8, 491.9, 492.0, 492.1, 492.2, 492.3, 492.4, 492.5, 492.6, 492.7, 492.8, 493.00, 493.01, 493.02, 493.03, 493.04, 493.05, 493.06, 493.07, 493.08, 493.09, 493.10, 493.11, 493.12, 493.13, 493.14, 493.15, 493.16, 493.17, 493.18, 493.19, 493.20, 493.21, 493.22, 493.23, 493.24, 493.25, 493.26, 493.27, 493.28, 493.29, 493.30, 493.31, 493.32, 493.33, 493.34, 493.35, 493.36, 493.37, 493.38, 493.39, 493.40, 493.41, 493.42, 493.43, 493.44, 493.45, 493.46, 493.47, 493.48, 493.49, 493.50, 493.51, 493.52, 493.53, 493.54, 493.55, 493.56, 493.57, 493.58, 493.59, 493.60, 493.61, 493.62, 493.63, 493.64, 493.65, 493.66, 493.67, 493.68, 493.69, 493.70, 493.71, 493.72, 493.73, 493.74, 493.75, 493.76, 493.77, 493.78, 493.79, 493.80, 493.81, 493.82, 493.83, 493.84, 493.85, 493.86, 493.87, 493.88, 493.89, 493.90, 493.91, 493.92, 494.X, 494.0, 494.1, 495.0, 495.1, 495.2, 495.3, 495.4, 495.5, 495.6, 495.7, 495.8, 495.9, 496.0, 496.1, 496.2, 496.3, 496.4, 496.5, 496.6, 496.7, 496.8, 496.9, 497.0, 497.1, 497.2, 497.3, 497.4, 497.5, 497.6, 497.7, 497.8, 497.9, 498.0, 498.1, 498.2, 498.3, 498.4, 498.5, 498.6, 498.7, 498.8, 498.9, 499.0, 499.1, 499.2, 499.3, 499.4, 499.5, 499.6, 499.7, 499.8, 499.9, 500.0, 500.1, 500.2, 500.3, 500.4, 500.5, 500.6, 500.7, 500.8, 500.9, 501.0, 501.1, 501.2, 501.3, 501.4, 501.5, 501.6, 501.7, 501.8, 501.9, 502.0, 502.1, 502.2, 502.3, 502.4, 502.5, 502.6, 502.7, 502.8, 502.9, 503.0, 503.1, 503.2, 503.3, 503.4, 503.5, 503.6, 503.7, 503.8, 503.9, 504.0, 504.1, 504.2, 504.3, 504.4, 504.5, 504.6, 504.7, 504.8, 504.9, 505.X, 506.4
Diabetes (uncomplicated)	E08.21, E08.22, E08.29, E08.311, E08.319, E08.321, E08.3211, E08.3212, E08.3213, E08.3219, E08.329, E08.3291, E08.3292, E08.3293, E08.3299, E08.331, E08.3311, E08.3312, E08.3313, E08.3319, E08.339, E08.3391, E08.3392, E08.3393, E08.3399, E08.341, E08.3411, E08.3412, E08.3413, E08.3419, E08.349, E08.3491, E08.3492, E08.3493, E08.3499, E08.351, E08.3511, E08.3512, E08.3513, E08.3519, E08.3521, E08.3522, E08.3523, E08.3529, E08.3531, E08.3532, E08.3533, E08.3539, E08.3541, E08.3542, E08.3543, E08.3549, E08.3551, E08.3552, E08.3553, E08.3559, E08.359, E08.3591, E08.3592, E08.3593, E08.3599, E08.36, E08.37X1, E08.37X2, E08.37X3, E08.37X9, E08.39, E08.40, E08.41, E08.42, E08.43, E08.44, E08.49, E08.51, E08.52, E08.59, E08.610, E08.618, E08.620, E08.621, E08.622, E08.628, E08.630, E08.638, E08.641, E08.649, E08.65, E08.69, E08.8, E09.21, E09.22, E09.29, E09.311, E09.319,

© 2022 Lapa ME et al. *JAMA Network Open.*

	E09.321, E09.3211, E09.3212, E09.3213, E09.3219, E09.329, E09.3291, E09.3292, E09.3293, E09.3299, E09.331, E09.3311, E09.3312, E09.3313, E09.3319, E09.339, E09.3391, E09.3392, E09.3393, E09.3399, E09.341, E09.3411, E09.3412, E09.3413, E09.3419, E09.349, E09.3491, E09.3492, E09.3493, E09.3499, E09.351, E09.3511, E09.3512, E09.3513, E09.3519, E09.3521, E09.3522, E09.3523, E09.3529, E09.3531, E09.3532, E09.3533, E09.3539, E09.3541, E09.3542, E09.3543, E09.3549, E09.3551, E09.3552, E09.3553, E09.3559, E09.359, E09.3591, E09.3592, E09.3593, E09.3599, E09.36, E09.37X1, E09.37X2, E09.37X3, E09.37X9, E09.39, E09.40, E09.41, E09.42, E09.43, E09.44, E09.49, E09.51, E09.52, E09.59, E09.610, E09.618, E09.620, E09.621, E09.622, E09.628, E09.630, E09.638, E09.641, E09.649, E09.65, E09.69, E09.8, E10.21, E10.22, E10.29, E10.311, E10.319, E10.321, E10.3211, E10.3212, E10.3213, E10.3219, E10.329, E10.3291, E10.3292, E10.3293, E10.3299, E10.331, E10.3311, E10.3312, E10.3313, E10.3319, E10.339, E10.3391, E10.3392, E10.3393, E10.3399, E10.341, E10.3411, E10.3412, E10.3413, E10.3419, E10.349, E10.3491, E10.3492, E10.3493, E10.3499, E10.351, E10.3511, E10.3512, E10.3513, E10.3519, E10.3521, E10.3522, E10.3523, E10.3529, E10.3531, E10.3532, E10.3533, E10.3539, E10.3541, E10.3542, E10.3543, E10.3549, E10.3551, E10.3552, E10.3553, E10.3559, E10.359, E10.3591, E10.3592, E10.3593, E10.3599, E10.36, E10.37X1, E10.37X2, E10.37X3, E10.37X9, E10.39, E10.40, E10.41, E10.42, E10.43, E10.44, E10.49, E10.51, E10.52, E10.59, E10.610, E10.618, E10.620, E10.621, E10.622, E10.628, E10.630, E10.638, E10.641, E10.649, E10.65, E10.69, E10.8, E11.21, E11.22, E11.29, E11.311, E11.319, E11.321, E11.3211, E11.3212, E11.3213, E11.3219, E11.329, E11.3291, E11.3292, E11.3293, E11.3299, E11.331, E11.3311, E11.3312, E11.3313, E11.3319, E11.339, E11.3391, E11.3392, E11.3393, E11.3399, E11.341, E11.3411, E11.3412, E11.3413, E11.3419, E11.349, E11.3491, E11.3492, E11.3493, E11.3499, E11.351, E11.3511, E11.3512, E11.3513, E11.3519, E11.3521, E11.3522, E11.3523, E11.3529, E11.3531, E11.3532, E11.3533, E11.3539, E11.3541, E11.3542, E11.3543, E11.3549, E11.3551, E11.3552, E11.3553, E11.3559, E11.359, E11.3591, E11.3592, E11.3593, E11.3599, E11.36, E11.37X1, E11.37X2, E11.37X3, E11.37X9, E11.39, E11.40, E11.41, E11.42, E11.43, E11.44, E11.49, E11.51, E11.52, E11.59, E11.610, E11.618, E11.620, E11.621, E11.622, E11.628, E11.630, E11.638, E11.641, E11.649, E11.65, E11.69, E11.8, E13.21, E13.22, E13.29, E13.311, E13.319, E13.321, E13.3211, E13.3212, E13.3213, E13.3219, E13.329, E13.3291, E13.3292, E13.3293, E13.3299, E13.331, E13.3311, E13.3312, E13.3313, E13.3319, E13.339, E13.3391, E13.3392, E13.3393, E13.3399, E13.341, E13.3411, E13.3412, E13.3413, E13.3419, E13.349, E13.3491, E13.3492, E13.3493, E13.3499, E13.351, E13.3511, E13.3512, E13.3513, E13.3519, E13.3521, E13.3522, E13.3523, E13.3529, E13.3531, E13.3532, E13.3533, E13.3539, E13.3541, E13.3542, E13.3543, E13.3549, E13.3551, E13.3552, E13.3553, E13.3559, E13.359, E13.3591, E13.3592, E13.3593, E13.3599, E13.36, E13.37X1, E13.37X2, E13.37X3, E13.37X9, E13.39, E13.40, E13.41, E13.42, E13.43, E13.44, E13.49, E13.51, E13.52, E13.59, E13.610, E13.618, E13.620, E13.621, E13.622, E13.628, E13.630, E13.638, E13.641, E13.649, E13.65, E13.69, E13.8, 249.00, 249.01, 249.02, 249.03, 249.04, 249.05, 249.06, 249.07, 249.08, 249.09, 249.10, 249.11, 249.12, 249.13, 249.14, 249.15, 249.16, 249.17, 249.18, 249.19, 249.20, 249.21, 249.22, 249.23, 249.24, 249.25, 249.26, 249.27, 249.28, 249.29, 249.30, 249.31, 250.00, 250.01, 250.02, 250.03, 250.04, 250.05, 250.06, 250.07, 250.08, 250.09, 250.10, 250.11, 250.12, 250.13, 250.14, 250.15, 250.16, 250.17, 250.18, 250.19, 250.20,

© 2022 Lapa ME et al. *JAMA Network Open.*

	250.21, 250.22, 250.23, 250.24, 250.25, 250.26, 250.27, 250.28, 250.29, 250.30, 250.31, 250.32, 250.33, 648.00, 648.01, 648.02, 648.03, 648.04
Diabetes (complicated)	E08.00, E08.01, E08.10, E08.11, E08.9, E09.00, E09.01, E09.10, E09.11, E09.9, E10.10, E10.11, E10.9, E11.00, E11.01, E11.10, E11.11, E11.9, E13.00, E13.01, E13.10, E13.11, E13.9, O24.011, O24.012, O24.013, O24.019, O24.02, O24.03, O24.111, O24.112, O24.113, O24.119, O24.12, O24.13, O24.311, O24.312, O24.313, O24.319, O24.32, O24.33, O24.410, O24.414, O24.415, O24.419, O24.420, O24.424, O24.425, O24.429, O24.430, O24.434, O24.435, O24.439, O24.811, O24.812, O24.813, O24.819, O24.82, O24.83, O24.911, O24.912, O24.913, O24.919, O24.92, O24.93, 249.40, 249.41, 249.42, 249.43, 249.44, 249.45, 249.46, 249.47, 249.48, 249.49, 249.50, 249.51, 249.52, 249.53, 249.54, 249.55, 249.56, 249.57, 249.58, 249.59, 249.60, 249.61, 249.62, 249.63, 249.64, 249.65, 249.66, 249.67, 249.68, 249.69, 249.70, 249.71, 249.72, 249.73, 249.74, 249.75, 249.76, 249.77, 249.78, 249.79, 249.80, 249.81, 249.82, 249.83, 249.84, 249.85, 249.86, 249.87, 249.88, 249.89, 249.90, 249.91, 250.40, 250.41, 250.42, 250.43, 250.44, 250.45, 250.46, 250.47, 250.48, 250.49, 250.50, 250.51, 250.52, 250.53, 250.54, 250.55, 250.56, 250.57, 250.58, 250.59, 250.60, 250.61, 250.62, 250.63, 250.64, 250.65, 250.66, 250.67, 250.68, 250.69, 250.70, 250.71, 250.72, 250.73, 250.74, 250.75, 250.76, 250.77, 250.78, 250.79, 250.80, 250.81, 250.82, 250.83, 250.84, 250.85, 250.86, 250.87, 250.88, 250.89, 250.90, 250.91, 250.92, 250.93, 775.1
Hypothyroidism	E00.0, E00.1, E00.2, E00.9, E01.0, E01.1, E01.2, E01.8, E02.X, E03.0, E03.1, E03.2, E03.3, E03.4, E03.5, E03.8, E03.9, E89.0, 243.0, 243.1, 243.2, 243.3, 243.4, 243.5, 243.6, 243.7, 243.8, 243.9, 244.0, 244.1, 244.2, 244.8, 244.9
Renal failure (moderate)	N18.3, N18.30, N18.31, N18.32, N18.9, N19.X, 403.01, 403.11, 403.91, 402.X, 404.03, 404.12, 404.13, 404.92, 404.93, 585.3, 585.4, 585.5, 585.6, 585.9, 586.X, V42.0, V45.1, V45.11, V45.12, V56.0, V56.00, V56.01, V56.02, V56.03, V56.04, V56.05, V56.06, V56.07, V56.08, V56.09, V56.10, V56.11, V56.12, V56.13, V56.14, V56.15, V56.16, V56.17, V56.18, V56.19, V56.20, V56.21, V56.22, V56.23, V56.24, V56.25, V56.26, V56.27, V56.28, V56.29, V56.30, V56.31, V56.32, V56.8
Renal failure (severe)	I12.0, I13.11, I13.2, N18.4, N18.5, N18.6, Z49.01, Z49.02, Z49.31, Z49.32, Z91.15, Z94.0, Z99.2
Liver disease (mild)	A51.45, A52.74, B18.0, B18.1, B18.2, B18.8, B18.9, B19.10, B19.20, B19.9, B25.1, B58.1, K70.0, K70.10, K70.11, K70.2, K70.30, K70.31, K70.9, K71.3, K71.4, K71.50, K71.51, K71.6, K71.7, K71.8, K73.0, K73.1, K73.2, K73.8, K73.9, K74.0, K74.00, K74.01, K74.02, K74.1, K74.2, K74.3, K74.4, K74.5, K74.60, K74.69, K75.1, K75.2, K75.3, K75.4, K75.81, K75.89, K75.9, K76.0, K76.1, K76.2, K76.3, K76.4, K76.81, K76.89, K76.9, K77.X, 702.2, 702.3, 703.2, 703.3, 704.4, 705.4, 456.0, 456.1, 456.20, 456.21, 571.0, 571.2, 571.3, 571.40, 571.41, 571.42, 571.43, 571.44, 571.45, 571.46, 571.47, 571.48, 571.49, 571.5, 571.6, 571.8, 571.9, 572.3, 572.8, 573.5, V42.7
Liver disease (moderate or severe)	B18.0, B18.1, B18.2, B18.8, B18.9, B19.0, B19.11, B19.21, B25.1, B58.1, I85.00, I85.01, I85.11, I86.4, K70.40, K70.41, K72.10, K72.11, K72.90, K76.5, K76.6, K76.7
Peptic ulcer disease (excluding bleeding)	K25.0, K25.1, K25.2, K25.3, K25.4, K25.5, K25.6, K25.7, K25.9, K26.0, K26.1, K26.2, K26.3, K26.4, K26.5, K26.6, K26.7, K26.9, K27.0, K27.1, K27.2, K27.3, K27.4, K27.5, K27.6, K27.7, K27.9, K28.0, K28.1, K28.2, K28.3, K28.4, K28.5, K28.6, K28.7, K28.9, 531.41, 531.51,

© 2022 Lapa ME et al. *JAMA Network Open.*

	531.61, 531.70, 531.71, 531.91, 532.41, 532.51, 532.61, 532.70, 532.71, 532.91, 533.41, 533.51, 533.61, 533.70, 533.71, 533.91, 534.41, 534.51, 534.61, 534.70, 534.71, 534.91
AIDS/HIV	B20.X, O98.711, O98.712, O98.713, O98.719, O98.72, O98.73, Z21.X, 042.X, 042.1, 042.2, 042.3, 042.4, 042.5, 042.6, 042.7, 042.8, 042.9, 043.0, 043.1, 043.2, 043.3, 043.4, 043.5, 043.6, 043.7, 043.8, 043.9, 044.0, 044.1, 044.2, 044.3, 044.4, 044.5, 044.6, 044.7, 044.8, 044.9, 043.X, 044.X
Lymphoma	C81.00, C81.01, C81.02, C81.03, C81.04, C81.05, C81.06, C81.07, C81.08, C81.09, C81.10, C81.11, C81.12, C81.13, C81.14, C81.15, C81.16, C81.17, C81.18, C81.19, C81.20, C81.21, C81.22, C81.23, C81.24, C81.25, C81.26, C81.27, C81.28, C81.29, C81.30, C81.31, C81.32, C81.33, C81.34, C81.35, C81.36, C81.37, C81.38, C81.39, C81.40, C81.41, C81.42, C81.43, C81.44, C81.45, C81.46, C81.47, C81.48, C81.49, C81.70, C81.71, C81.72, C81.73, C81.74, C81.75, C81.76, C81.77, C81.78, C81.79, C81.90, C81.91, C81.92, C81.93, C81.94, C81.95, C81.96, C81.97, C81.98, C81.99, C82.00, C82.01, C82.02, C82.03, C82.04, C82.05, C82.06, C82.07, C82.08, C82.09, C82.10, C82.11, C82.12, C82.13, C82.14, C82.15, C82.16, C82.17, C82.18, C82.19, C82.20, C82.21, C82.22, C82.23, C82.24, C82.25, C82.26, C82.27, C82.28, C82.29, C82.30, C82.31, C82.32, C82.33, C82.34, C82.35, C82.36, C82.37, C82.38, C82.39, C82.40, C82.41, C82.42, C82.43, C82.44, C82.45, C82.46, C82.47, C82.48, C82.49, C82.50, C82.51, C82.52, C82.53, C82.54, C82.55, C82.56, C82.57, C82.58, C82.59, C82.60, C82.61, C82.62, C82.63, C82.64, C82.65, C82.66, C82.67, C82.68, C82.69, C82.80, C82.81, C82.82, C82.83, C82.84, C82.85, C82.86, C82.87, C82.88, C82.89, C82.90, C82.91, C82.92, C82.93, C82.94, C82.95, C82.96, C82.97, C82.98, C82.99, C83.00, C83.01, C83.02, C83.03, C83.04, C83.05, C83.06, C83.07, C83.08, C83.09, C83.10, C83.11, C83.12, C83.13, C83.14, C83.15, C83.16, C83.17, C83.18, C83.19, C83.30, C83.31, C83.32, C83.33, C83.34, C83.35, C83.36, C83.37, C83.38, C83.39, C83.50, C83.51, C83.52, C83.53, C83.54, C83.55, C83.56, C83.57, C83.58, C83.59, C83.70, C83.71, C83.72, C83.73, C83.74, C83.75, C83.76, C83.77, C83.78, C83.79, C83.80, C83.81, C83.82, C83.83, C83.84, C83.85, C83.86, C83.87, C83.88, C83.89, C83.90, C83.91, C83.92, C83.93, C83.94, C83.95, C83.96, C83.97, C83.98, C83.99, C84.00, C84.01, C84.02, C84.03, C84.04, C84.05, C84.06, C84.07, C84.08, C84.09, C84.10, C84.11, C84.12, C84.13, C84.14, C84.15, C84.16, C84.17, C84.18, C84.19, C84.40, C84.41, C84.42, C84.43, C84.44, C84.45, C84.46, C84.47, C84.48, C84.49, C84.60, C84.61, C84.62, C84.63, C84.64, C84.65, C84.66, C84.67, C84.68, C84.69, C84.70, C84.71, C84.72, C84.73, C84.74, C84.75, C84.76, C84.77, C84.78, C84.79, C84.90, C84.91, C84.92, C84.93, C84.94, C84.95, C84.96, C84.97, C84.98, C84.99, C84.A0, C84.A1, C84.A2, C84.A3, C84.A4, C84.A5, C84.A6, C84.A7, C84.A8, C84.A9, C84.Z0, C84.Z1, C84.Z2, C84.Z3, C84.Z4, C84.Z5, C84.Z6, C84.Z7, C84.Z8, C84.Z9, C85.10, C85.11, C85.12, C85.13, C85.14, C85.15, C85.16, C85.17, C85.18, C85.19, C85.20, C85.21, C85.22, C85.23, C85.24, C85.25, C85.26, C85.27, C85.28, C85.29, C85.80, C85.81, C85.82, C85.83, C85.84, C85.85, C85.86, C85.87, C85.88, C85.89, C85.90, C85.91, C85.92, C85.93, C85.94, C85.95, C85.96, C85.97, C85.98, C85.99, C86.0, C86.1, C86.2, C86.3, C86.4, C86.5, C86.6, C88.0, C88.2, C88.3, C88.4, C88.8, C88.9, C90.00, C90.01, C90.02, C90.20, C90.21, C90.22, C90.30, C90.31, C90.32, C96.0, C96.2, C96.20, C96.21, C96.22, C96.29, C96.4, C96.9, C96.A, C96.Z, D47.Z9, 200.00, 200.01, 200.02, 200.03, 200.04, 200.05, 200.06, 200.07, 200.08, 200.09, 200.10, 200.11, 200.12, 200.13, 200.14, 200.15, 200.16, 200.17, 200.18, 200.19, 200.20, 200.21, 200.22, 200.23,

© 2022 Lapa ME et al. *JAMA Network Open.*

	200.24, 200.25, 200.26, 200.27, 200.28, 200.29, 200.30, 200.31, 200.32, 200.33, 200.34, 200.35, 200.36, 200.37, 200.38, 200.39, 200.40, 200.41, 200.42, 200.43, 200.44, 200.45, 200.46, 200.47, 200.48, 200.49, 200.50, 200.51, 200.52, 200.53, 200.54, 200.55, 200.56, 200.57, 200.58, 200.59, 200.60, 200.61, 200.62, 200.63, 200.64, 200.65, 200.66, 200.67, 200.68, 200.69, 200.70, 200.71, 200.72, 200.73, 200.74, 200.75, 200.76, 200.77, 200.78, 200.79, 200.80, 200.81, 200.82, 200.83, 200.84, 200.85, 200.86, 200.87, 200.88, 200.89, 200.90, 200.91, 200.92, 200.93, 200.94, 200.95, 200.96, 200.97, 200.98, 200.99, 201.00, 201.01, 201.02, 201.03, 201.04, 201.05, 201.06, 201.07, 201.08, 201.09, 201.10, 201.11, 201.12, 201.13, 201.14, 201.15, 201.16, 201.17, 201.18, 201.19, 201.20, 201.21, 201.22, 201.23, 201.24, 201.25, 201.26, 201.27, 201.28, 201.29, 201.30, 201.31, 201.32, 201.33, 201.34, 201.35, 201.36, 201.37, 201.38, 201.39, 201.40, 201.41, 201.42, 201.43, 201.44, 201.45, 201.46, 201.47, 201.48, 201.49, 201.50, 201.51, 201.52, 201.53, 201.54, 201.55, 201.56, 201.57, 201.58, 201.59, 201.60, 201.61, 201.62, 201.63, 201.64, 201.65, 201.66, 201.67, 201.68, 201.69, 201.70, 201.71, 201.72, 201.73, 201.74, 201.75, 201.76, 201.77, 201.78, 201.79, 201.80, 201.81, 201.82, 201.83, 201.84, 201.85, 201.86, 201.87, 201.88, 201.89, 201.90, 201.91, 201.92, 201.93, 201.94, 201.95, 201.96, 201.97, 201.98, 201.99, 202.00, 202.01, 202.02, 202.03, 202.04, 202.05, 202.06, 202.07, 202.08, 202.09, 202.10, 202.11, 202.12, 202.13, 202.14, 202.15, 202.16, 202.17, 202.18, 202.19, 202.20, 202.21, 202.22, 202.23, 202.24, 202.25, 202.26, 202.27, 202.28, 202.29, 202.30, 202.31, 202.32, 202.33, 202.34, 202.35, 202.36, 202.37, 202.38, 202.50, 202.51, 202.52, 202.53, 202.54, 202.55, 202.56, 202.57, 202.58, 202.59, 202.60, 202.61, 202.62, 202.63, 202.64, 202.65, 202.66, 202.67, 202.68, 202.69, 202.70, 202.71, 202.72, 202.73, 202.74, 202.75, 202.76, 202.77, 202.78, 202.79, 202.80, 202.81, 202.82, 202.83, 202.84, 202.85, 202.86, 202.87, 202.88, 202.89, 202.90, 202.91, 202.92, 202.93, 202.94, 202.95, 202.96, 202.97, 202.98, 202.99, 203.00, 203.01, 203.02, 203.03, 203.04, 203.05, 203.06, 203.07, 203.08, 203.09, 203.10, 203.11, 203.12, 203.13, 203.14, 203.15, 203.16, 203.17, 203.18, 203.19, 203.20, 203.21, 203.22, 203.23, 203.24, 203.25, 203.26, 203.27, 203.28, 203.29, 203.30, 203.31, 203.32, 203.33, 203.34, 203.35, 203.36, 203.37, 203.38, 203.39, 203.40, 203.41, 203.42, 203.43, 203.44, 203.45, 203.46, 203.47, 203.48, 203.49, 203.50, 203.51, 203.52, 203.53, 203.54, 203.55, 203.56, 203.57, 203.58, 203.59, 203.60, 203.61, 203.62, 203.63, 203.64, 203.65, 203.66, 203.67, 203.68, 203.69, 203.70, 203.71, 203.72, 203.73, 203.74, 203.75, 203.76, 203.77, 203.78, 203.79, 203.80, 203.81, 238.6, 273.3
Leukemia	C90.10, C90.11, C90.12, C91.00, C91.01, C91.02, C91.10, C91.11, C91.12, C91.30, C91.31, C91.32, C91.40, C91.41, C91.42, C91.50, C91.51, C91.52, C91.60, C91.61, C91.62, C91.90, C91.91, C91.92, C91.A0, C91.A1, C91.A2, C91.Z0, C91.Z1, C91.Z2, C92.00, C92.01, C92.02, C92.10, C92.11, C92.12, C92.20, C92.21, C92.22, C92.30, C92.31, C92.32, C92.40, C92.41, C92.42, C92.50, C92.51, C92.52, C92.60, C92.61, C92.62, C92.90, C92.91, C92.92, C92.A0, C92.A1, C92.A2, C92.Z0, C92.Z1, C92.Z2, C93.00, C93.01, C93.02, C93.10, C93.11, C93.12, C93.30, C93.31, C93.32, C93.90, C93.91, C93.92, C93.Z0, C93.Z1, C93.Z2, C94.00, C94.01, C94.02, C94.20, C94.21, C94.22, C94.30, C94.31, C94.32, C94.40, C94.41, C94.42, C94.6, C94.80, C94.81, C94.82, C95.00, C95.01, C95.02, C95.10, C95.11, C95.12, C95.90, C95.91, C95.92
Cancer (in situ)	D00.00, D00.01, D00.02, D00.03, D00.04, D00.05, D00.06, D00.07, D00.08, D00.1, D00.2, D01.0, D01.1, D01.2, D01.3, D01.40, D01.49, D01.5, D01.7, D01.9, D02.0, D02.1, D02.20, D02.21, D02.22, D02.3, D02.4, D03.0, D03.10, D03.11, D03.111, D03.112, D03.12, D03.121, D03.122, D03.20, D03.21, D03.22, D03.30, D03.39, D03.4, D03.51, D03.52,

© 2022 Lapa ME et al. *JAMA Network Open.*

	D03.59, D03.60, D03.61, D03.62, D03.70, D03.71, D03.72, D03.8, D03.9, D04.0, D04.10, D04.11, D04.111, D04.112, D04.12, D04.121, D04.122, D04.20, D04.21, D04.22, D04.30, D04.39, D04.4, D04.5, D04.60, D04.61, D04.62, D04.70, D04.71, D04.72, D04.8, D04.9, D05.00, D05.01, D05.02, D05.10, D05.11, D05.12, D05.80, D05.81, D05.82, D05.90, D05.91, D05.92, D06.0, D06.1, D06.7, D06.9, D07.0, D07.1, D07.2, D07.30, D07.39, D07.4, D07.5, D07.60, D07.61, D07.69, D09.0, D09.10, D09.19, D09.20, D09.21, D09.22, D09.3, D09.8, D09.9, 140.0, 140.1, 140.2, 140.3, 140.4, 140.5, 140.6, 140.7, 140.8, 140.9, 141.0, 141.1, 141.2, 141.3, 141.4, 141.5, 141.6, 141.7, 141.8, 141.9, 142.0, 142.1, 142.2, 142.3, 142.4, 142.5, 142.6, 142.7, 142.8, 142.9, 143.0, 143.1, 143.2, 143.3, 143.4, 143.5, 143.6, 143.7, 143.8, 143.9, 144.0, 144.1, 144.2, 144.3, 144.4, 144.5, 144.6, 144.7, 144.8, 144.9, 145.0, 145.1, 145.2, 145.3, 145.4, 145.5, 145.6, 145.7, 145.8, 145.9, 146.0, 146.1, 146.2, 146.3, 146.4, 146.5, 146.6, 146.7, 146.8, 146.9, 147.0, 147.1, 147.2, 147.3, 147.4, 147.5, 147.6, 147.7, 147.8, 147.9, 148.0, 148.1, 148.2, 148.3, 148.4, 148.5, 148.6, 148.7, 148.8, 148.9, 149.0, 149.1, 149.2, 149.3, 149.4, 149.5, 149.6, 149.7, 149.8, 149.9, 150.0, 150.1, 150.2, 150.3, 150.4, 150.5, 150.6, 150.7, 150.8, 150.9, 151.0, 151.1, 151.2, 151.3, 151.4, 151.5, 151.6, 151.7, 151.8, 151.9, 152.0, 152.1, 152.2, 152.3, 152.4, 152.5, 152.6, 152.7, 152.8, 152.9, 153.0, 153.1, 153.2, 153.3, 153.4, 153.5, 153.6, 153.7, 153.8, 153.9, 154.0, 154.1, 154.2, 154.3, 154.4, 154.5, 154.6, 154.7, 154.8, 154.9, 155.0, 155.1, 155.2, 155.3, 155.4, 155.5, 155.6, 155.7, 155.8, 155.9, 156.0, 156.1, 156.2, 156.3, 156.4, 156.5, 156.6, 156.7, 156.8, 156.9, 157.0, 157.1, 157.2, 157.3, 157.4, 157.5, 157.6, 157.7, 157.8, 157.9, 158.0, 158.1, 158.2, 158.3, 158.4, 158.5, 158.6, 158.7, 158.8, 158.9, 159.0, 159.1, 159.2, 159.3, 159.4, 159.5, 159.6, 159.7, 159.8, 159.9, 160.0, 160.1, 160.2, 160.3, 160.4, 160.5, 160.6, 160.7, 160.8, 160.9, 161.0, 161.1, 161.2, 161.3, 161.4, 161.5, 161.6, 161.7, 161.8, 161.9, 162.0, 162.1, 162.2, 162.3, 162.4, 162.5, 162.6, 162.7, 162.8, 162.9, 163.0, 163.1, 163.2, 163.3, 163.4, 163.5, 163.6, 163.7, 163.8, 163.9, 164.0, 164.1, 164.2, 164.3, 164.4, 164.5, 164.6, 164.7, 164.8, 164.9, 165.0, 165.1, 165.2, 165.3, 165.4, 165.5, 165.6, 165.7, 165.8, 165.9, 166.0, 166.1, 166.2, 166.3, 166.4, 166.5, 166.6, 166.7, 166.8, 166.9, 167.0, 167.1, 167.2, 167.3, 167.4, 167.5, 167.6, 167.7, 167.8, 167.9, 168.0, 168.1, 168.2, 168.3, 168.4, 168.5, 168.6, 168.7, 168.8, 168.9, 169.0, 169.1, 169.2, 169.3, 169.4, 169.5, 169.6, 169.7, 169.8, 169.9, 170.0, 170.1, 170.2, 170.3, 170.4, 170.5, 170.6, 170.7, 170.8, 170.9, 171.0, 171.1, 171.2, 171.3, 171.4, 171.5, 171.6, 171.7, 171.8, 171.9, 172.0, 172.1, 172.2, 172.3, 172.4, 172.5, 172.6, 172.7, 172.8, 172.9, 174.0, 174.1, 174.2, 174.3, 174.4, 174.5, 174.6, 174.7, 174.8, 174.9, 175.0, 175.1, 175.2, 175.3, 175.4, 175.5, 175.6, 175.7, 175.8, 175.9, 179.0, 179.1, 179.2, 179.3, 179.4, 179.5, 179.6, 179.7, 179.8, 179.9, 180.0, 180.1, 180.2, 180.3, 180.4, 180.5, 180.6, 180.7, 180.8, 180.9, 181.0, 181.1, 181.2, 181.3, 181.4, 181.5, 181.6, 181.7, 181.8, 181.9, 182.0, 182.1, 182.2, 182.3, 182.4, 182.5, 182.6, 182.7, 182.8, 182.9, 183.0, 183.1, 183.2, 183.3, 183.4, 183.5, 183.6, 183.7, 183.8, 183.9, 184.0, 184.1, 184.2, 184.3, 184.4, 184.5, 184.6, 184.7, 184.8, 184.9, 185.0, 185.1, 185.2, 185.3, 185.4, 185.5, 185.6, 185.7, 185.8, 185.9, 186.0, 186.1, 186.2, 186.3, 186.4, 186.5, 186.6, 186.7, 186.8, 186.9, 187.0, 187.1, 187.2, 187.3, 187.4, 187.5, 187.6, 187.7, 187.8, 187.9, 188.0, 188.1, 188.2, 188.3, 188.4, 188.5, 188.6, 188.7, 188.8, 188.9, 189.0, 189.1, 189.2, 189.3, 189.4, 189.5, 189.6, 189.7, 189.8, 189.9, 190.0, 190.1, 190.2, 190.3, 190.4, 190.5, 190.6, 190.7, 190.8, 190.9, 191.0, 191.1, 191.2, 191.3, 191.4, 191.5, 191.6, 191.7, 191.8, 191.9, 192.0, 192.1, 192.2, 192.3, 192.4, 192.5, 192.6, 192.7, 192.8, 192.9, 193.0, 193.1, 193.2, 193.3, 193.4, 193.5, 193.6, 193.7, 193.8, 193.9, 194.0, 194.1, 194.2, 194.3, 194.4, 194.5, 194.6, 194.7, 194.8, 194.9, 195.0, 195.1, 195.2, 195.3, 195.4, 195.5, 195.6, 195.7, 195.8, 209.00, 209.01, 209.02, 209.03, 209.04, 209.05, 209.06, 209.07, 209.08, 209.09,

	209.10, 209.11, 209.12, 209.13, 209.14, 209.15, 209.16, 209.17, 209.18, 209.19, 209.20, 209.21, 209.22, 209.23, 209.24, 209.25, 209.26, 209.27, 209.28, 209.29, 209.3, 209.30, 209.31, 209.32, 209.33, 209.34, 209.35, 209.36, 258.01, 258.02, 258.03
Metastatic cancer	C77.0, C77.1, C77.2, C77.3, C77.4, C77.5, C77.8, C77.9, C78.00, C78.01, C78.02, C78.1, C78.2, C78.30, C78.39, C78.4, C78.5, C78.6, C78.7, C78.80, C78.89, C79.00, C79.01, C79.02, C79.10, C79.11, C79.19, C79.2, C79.31, C79.32, C79.40, C79.49, C79.51, C79.52, C79.60, C79.61, C79.62, C79.70, C79.71, C79.72, C79.81, C79.82, C79.89, C79.9, C7B.00, C7B.01, C7B.02, C7B.03, C7B.04, C7B.09, C7B.1, C7B.8, C80.0, 196.0, 196.1, 196.2, 196.3, 196.4, 196.5, 196.6, 196.7, 196.8, 196.9, 197.0, 197.1, 197.2, 197.3, 197.4, 197.5, 197.6, 197.7, 197.8, 197.9, 198.0, 198.1, 198.2, 198.3, 198.4, 198.5, 198.6, 198.7, 198.8, 198.9, 199.0, 199.1, 209.70, 209.71, 209.72, 209.73, 209.74, 209.75, 209.79, 789.51
Solid tumor w/o metastasis (malignant)	C00.0, C00.1, C00.2, C00.3, C00.4, C00.5, C00.6, C00.8, C00.9, C01.X, C02.0, C02.1, C02.2, C02.3, C02.4, C02.8, C02.9, C03.0, C03.1, C03.9, C04.0, C04.1, C04.8, C04.9, C05.0, C05.1, C05.2, C05.8, C05.9, C06.0, C06.1, C06.2, C06.80, C06.89, C06.9, C07.X, C08.0, C08.1, C08.9, C09.0, C09.1, C09.8, C09.9, C10.0, C10.1, C10.2, C10.3, C10.4, C10.8, C10.9, C11.0, C11.1, C11.2, C11.3, C11.8, C11.9, C12.X, C13.0, C13.1, C13.2, C13.8, C13.9, C14.0, C14.2, C14.8, C15.3, C15.4, C15.5, C15.8, C15.9, C16.0, C16.1, C16.2, C16.3, C16.4, C16.5, C16.6, C16.8, C16.9, C17.0, C17.1, C17.2, C17.3, C17.8, C17.9, C18.0, C18.1, C18.2, C18.3, C18.4, C18.5, C18.6, C18.7, C18.8, C18.9, C19.X, C20.X, C21.0, C21.1, C21.2, C21.8, C22.0, C22.1, C22.2, C22.3, C22.4, C22.7, C22.8, C22.9, C23.X, C24.0, C24.1, C24.8, C24.9, C25.0, C25.1, C25.2, C25.3, C25.4, C25.7, C25.8, C25.9, C26.0, C26.1, C26.9, C30.0, C30.1, C31.0, C31.1, C31.2, C31.3, C31.8, C31.9, C32.0, C32.1, C32.2, C32.3, C32.8, C32.9, C33.X, C34.00, C34.01, C34.02, C34.10, C34.11, C34.12, C34.2, C34.30, C34.31, C34.32, C34.80, C34.81, C34.82, C34.90, C34.91, C34.92, C37.X, C38.0, C38.1, C38.2, C38.3, C38.4, C38.8, C39.0, C39.9, C40.00, C40.01, C40.02, C40.10, C40.11, C40.12, C40.20, C40.21, C40.22, C40.30, C40.31, C40.32, C40.80, C40.81, C40.82, C40.90, C40.91, C40.92, C41.0, C41.1, C41.2, C41.3, C41.4, C41.9, C43.0, C43.10, C43.11, C43.111, C43.112, C43.12, C43.121, C43.122, C43.20, C43.21, C43.22, C43.30, C43.31, C43.39, C43.4, C43.51, C43.52, C43.59, C43.60, C43.61, C43.62, C43.70, C43.71, C43.72, C43.8, C43.9, C44.00, C44.09, C44.101, C44.102, C44.1021, C44.1022, C44.109, C44.1091, C44.1092, C44.131, C44.1321, C44.1322, C44.1391, C44.1392, C44.191, C44.192, C44.1921, C44.1922, C44.199, C44.1991, C44.1992, C44.201, C44.202, C44.209, C44.291, C44.292, C44.299, C44.300, C44.301, C44.309, C44.390, C44.391, C44.399, C44.40, C44.49, C44.500, C44.501, C44.509, C44.590, C44.591, C44.599, C44.601, C44.602, C44.609, C44.691, C44.692, C44.699, C44.701, C44.702, C44.709, C44.791, C44.792, C44.799, C44.80, C44.89, C44.90, C44.99, C45.0, C45.1, C45.2, C45.7, C45.9, C46.0, C46.1, C46.2, C46.3, C46.4, C46.50, C46.51, C46.52, C46.7, C46.9, C47.0, C47.10, C47.11, C47.12, C47.20, C47.21, C47.22, C47.3, C47.4, C47.5, C47.6, C47.8, C47.9, C48.0, C48.1, C48.2, C48.8, C49.0, C49.10, C49.11, C49.12, C49.20, C49.21, C49.22, C49.3, C49.4, C49.5, C49.6, C49.8, C49.9, C49.A0, C49.A1, C49.A2, C49.A3, C49.A4, C49.A5, C49.A9, C4A.0, C4A.10, C4A.11, C4A.111, C4A.112, C4A.12, C4A.121, C4A.122, C4A.20, C4A.21, C4A.22, C4A.30, C4A.31, C4A.39, C4A.4, C4A.51, C4A.52, C4A.59, C4A.60, C4A.61, C4A.62, C4A.70, C4A.71, C4A.72, C4A.8, C4A.9, C50.011, C50.012, C50.019, C50.021, C50.022, C50.029, C50.111, C50.112, C50.119, C50.121, C50.122, C50.129, C50.211, C50.212, C50.219, C50.221, C50.222, C50.229, C50.311, C50.312, C50.319, C50.321, C50.322, C50.329, C50.411, C50.412,

© 2022 Lapa ME et al. *JAMA Network Open.*

	C50.419, C50.421, C50.422, C50.429, C50.511, C50.512, C50.519, C50.521, C50.522, C50.529, C50.611, C50.612, C50.619, C50.621, C50.622, C50.629, C50.811, C50.812, C50.819, C50.821, C50.822, C50.829, C50.911, C50.912, C50.919, C50.921, C50.922, C50.929, C51.0, C51.1, C51.2, C51.8, C51.9, C52.X, C53.0, C53.1, C53.8, C53.9, C54.0, C54.1, C54.2, C54.3, C54.8, C54.9, C55.X, C56.1, C56.2, C56.9, C57.00, C57.01, C57.02, C57.10, C57.11, C57.12, C57.20, C57.21, C57.22, C57.3, C57.4, C57.7, C57.8, C57.9, C58.X, C60.0, C60.1, C60.2, C60.8, C60.9, C61.X, C62.00, C62.01, C62.02, C62.10, C62.11, C62.12, C62.90, C62.91, C62.92, C63.00, C63.01, C63.02, C63.10, C63.11, C63.12, C63.2, C63.7, C63.8, C63.9, C64.1, C64.2, C64.9, C65.1, C65.2, C65.9, C66.1, C66.2, C66.9, C67.0, C67.1, C67.2, C67.3, C67.4, C67.5, C67.6, C67.7, C67.8, C67.9, C68.0, C68.1, C68.8, C68.9, C69.00, C69.01, C69.02, C69.10, C69.11, C69.12, C69.20, C69.21, C69.22, C69.30, C69.31, C69.32, C69.40, C69.41, C69.42, C69.50, C69.51, C69.52, C69.60, C69.61, C69.62, C69.80, C69.81, C69.82, C69.90, C69.91, C69.92, C70.0, C70.1, C70.9, C71.0, C71.1, C71.2, C71.3, C71.4, C71.5, C71.6, C71.7, C71.8, C71.9, C72.0, C72.1, C72.20, C72.21, C72.22, C72.30, C72.31, C72.32, C72.40, C72.41, C72.42, C72.50, C72.59, C72.9, C73.X, C74.00, C74.01, C74.02, C74.10, C74.11, C74.12, C74.90, C74.91, C74.92, C75.0, C75.1, C75.2, C75.3, C75.4, C75.5, C75.8, C75.9, C76.0, C76.1, C76.2, C76.3, C76.40, C76.41, C76.42, C76.50, C76.51, C76.52, C76.8, C7A.00, C7A.010, C7A.011, C7A.012, C7A.019, C7A.020, C7A.021, C7A.022, C7A.023, C7A.024, C7A.025, C7A.026, C7A.029, C7A.090, C7A.091, C7A.092, C7A.093, C7A.094, C7A.095, C7A.096, C7A.098, C7A.1, C7A.8, D46.9, E31.21, E31.22, E31.23
Arthropathies	L40.50, L40.51, L40.54, L40.59, L90.0, L94.0, L94.1, L94.3, M01.X0, M01.X11, M01.X12, M01.X19, M01.X21, M01.X22, M01.X29, M01.X31, M01.X32, M01.X39, M01.X41, M01.X42, M01.X49, M01.X51, M01.X52, M01.X59, M01.X61, M01.X62, M01.X69, M01.X71, M01.X72, M01.X79, M01.X8, M01.X9, M02.00, M02.011, M02.012, M02.019, M02.021, M02.022, M02.029, M02.031, M02.032, M02.039, M02.041, M02.042, M02.049, M02.051, M02.052, M02.059, M02.061, M02.062, M02.069, M02.071, M02.072, M02.079, M02.08, M02.09, M02.10, M02.111, M02.112, M02.119, M02.121, M02.122, M02.129, M02.131, M02.132, M02.139, M02.141, M02.142, M02.149, M02.151, M02.152, M02.159, M02.161, M02.162, M02.169, M02.171, M02.172, M02.179, M02.18, M02.19, M02.20, M02.211, M02.212, M02.219, M02.221, M02.222, M02.229, M02.231, M02.232, M02.239, M02.241, M02.242, M02.249, M02.251, M02.252, M02.259, M02.261, M02.262, M02.269, M02.271, M02.272, M02.279, M02.28, M02.29, M02.30, M02.311, M02.312, M02.319, M02.321, M02.322, M02.329, M02.331, M02.332, M02.339, M02.341, M02.342, M02.349, M02.351, M02.352, M02.359, M02.361, M02.362, M02.369, M02.371, M02.372, M02.379, M02.38, M02.39, M02.80, M02.811, M02.812, M02.819, M02.821, M02.822, M02.829, M02.831, M02.832, M02.839, M02.841, M02.842, M02.849, M02.851, M02.852, M02.859, M02.861, M02.862, M02.869, M02.871, M02.872, M02.879, M02.88, M02.89, M02.9, M05.00, M05.011, M05.012, M05.019, M05.021, M05.022, M05.029, M05.031, M05.032, M05.039, M05.041, M05.042, M05.049, M05.051, M05.052, M05.059, M05.061, M05.062, M05.069, M05.071, M05.072, M05.079, M05.09, M05.10, M05.111, M05.112, M05.119, M05.121, M05.122, M05.129, M05.131, M05.132, M05.139, M05.141, M05.142, M05.149, M05.151, M05.152, M05.159, M05.161, M05.162, M05.169, M05.171, M05.172, M05.179, M05.19, M05.20, M05.211, M05.212, M05.219, M05.221, M05.222, M05.229, M05.231, M05.232, M05.239, M05.241, M05.242, M05.249, M05.251, M05.252, M05.259, M05.261, M05.262, M05.269, M05.271, M05.272, M05.279, M05.29, M05.30, M05.311, M05.312, M05.319, M05.321,

© 2022 Lapa ME et al. *JAMA Network Open.*

	M05.322, M05.329, M05.331, M05.332, M05.339, M05.341, M05.342, M05.349, M05.351, M05.352, M05.359, M05.361, M05.362, M05.369, M05.371, M05.372, M05.379, M05.39, M05.40, M05.411, M05.412, M05.419, M05.421, M05.422, M05.429, M05.431, M05.432, M05.439, M05.441, M05.442, M05.449, M05.451, M05.452, M05.459, M05.461, M05.462, M05.469, M05.471, M05.472, M05.479, M05.49, M05.50, M05.511, M05.512, M05.519, M05.521, M05.522, M05.529, M05.531, M05.532, M05.539, M05.541, M05.542, M05.549, M05.551, M05.552, M05.559, M05.561, M05.562, M05.569, M05.571, M05.572, M05.579, M05.59, M05.60, M05.611, M05.612, M05.619, M05.621, M05.622, M05.629, M05.631, M05.632, M05.639, M05.641, M05.642, M05.649, M05.651, M05.652, M05.659, M05.661, M05.662, M05.669, M05.671, M05.672, M05.679, M05.69, M05.70, M05.711, M05.712, M05.719, M05.721, M05.722, M05.729, M05.731, M05.732, M05.739, M05.741, M05.742, M05.749, M05.751, M05.752, M05.759, M05.761, M05.762, M05.769, M05.771, M05.772, M05.779, M05.79, M05.7A, M05.80, M05.811, M05.812, M05.819, M05.821, M05.822, M05.829, M05.831, M05.832, M05.839, M05.841, M05.842, M05.849, M05.851, M05.852, M05.859, M05.861, M05.862, M05.869, M05.871, M05.872, M05.879, M05.89, M05.8A, M05.9, M06.00, M06.011, M06.012, M06.019, M06.021, M06.022, M06.029, M06.031, M06.032, M06.039, M06.041, M06.042, M06.049, M06.051, M06.052, M06.059, M06.061, M06.062, M06.069, M06.071, M06.072, M06.079, M06.08, M06.09, M06.0A, M06.1, M06.20, M06.211, M06.212, M06.219, M06.221, M06.222, M06.229, M06.231, M06.232, M06.239, M06.241, M06.242, M06.249, M06.251, M06.252, M06.259, M06.261, M06.262, M06.269, M06.271, M06.272, M06.279, M06.28, M06.29, M06.30, M06.311, M06.312, M06.319, M06.321, M06.322, M06.329, M06.331, M06.332, M06.339, M06.341, M06.342, M06.349, M06.351, M06.352, M06.359, M06.361, M06.362, M06.369, M06.371, M06.372, M06.379, M06.38, M06.39, M06.4, M06.80, M06.811, M06.812, M06.819, M06.821, M06.822, M06.829, M06.831, M06.832, M06.839, M06.841, M06.842, M06.849, M06.851, M06.852, M06.859, M06.861, M06.862, M06.869, M06.871, M06.872, M06.879, M06.88, M06.89, M06.8A, M06.9, M07.60, M07.611, M07.612, M07.619, M07.621, M07.622, M07.629, M07.631, M07.632, M07.639, M07.641, M07.642, M07.649, M07.651, M07.652, M07.659, M07.661, M07.662, M07.669, M07.671, M07.672, M07.679, M07.68, M07.69, M08.00, M08.011, M08.012, M08.019, M08.021, M08.022, M08.029, M08.031, M08.032, M08.039, M08.041, M08.042, M08.049, M08.051, M08.052, M08.059, M08.061, M08.062, M08.069, M08.071, M08.072, M08.079, M08.08, M08.09, M08.0A, M08.1, M08.20, M08.211, M08.212, M08.219, M08.221, M08.222, M08.229, M08.231, M08.232, M08.239, M08.241, M08.242, M08.249, M08.251, M08.252, M08.259, M08.261, M08.262, M08.269, M08.271, M08.272, M08.279, M08.28, M08.29, M08.2A, M08.3, M08.40, M08.411, M08.412, M08.419, M08.421, M08.422, M08.429, M08.431, M08.432, M08.439, M08.441, M08.442, M08.449, M08.451, M08.452, M08.459, M08.461, M08.462, M08.469, M08.471, M08.472, M08.479, M08.48, M08.4A, M08.80, M08.811, M08.812, M08.819, M08.821, M08.822, M08.829, M08.831, M08.832, M08.839, M08.841, M08.842, M08.849, M08.851, M08.852, M08.859, M08.861, M08.862, M08.869, M08.871, M08.872, M08.879, M08.88, M08.89, M08.90, M08.911, M08.912, M08.919, M08.921, M08.922, M08.929, M08.931, M08.932, M08.939, M08.941, M08.942, M08.949, M08.951, M08.952, M08.959, M08.961, M08.962, M08.969, M08.971, M08.972, M08.979, M08.98, M08.99, M08.9A, M12.00, M12.011, M12.012, M12.019, M12.021, M12.022, M12.029, M12.031, M12.032, M12.039, M12.041, M12.042, M12.049, M12.051, M12.052, M12.059, M12.061, M12.062, M12.069, M12.071, M12.072, M12.079, M12.08, M12.09, M30.0, M30.1, M30.2, M30.3, M30.8, M31.0, M31.1, M31.2, M31.30, M31.31, M31.4, M31.5, M31.6, M31.7, M31.8, M31.9, M32.0, M32.10, M32.12, M32.13,

© 2022 Lapa ME et al. *JAMA Network Open.*

	M32.14, M32.15, M32.19, M32.8, M32.9, M33.00, M33.01, M33.02, M33.03, M33.09, M33.10, M33.11, M33.12, M33.13, M33.19, M33.20, M33.21, M33.22, M33.29, M33.90, M33.91, M33.92, M33.93, M33.99, M34.0, M34.1, M34.2, M34.81, M34.82, M34.83, M34.89, M34.9, M35.00, M35.01, M35.02, M35.03, M35.04, M35.09, M35.1, M35.2, M35.3, M35.5, M35.6, M35.8, M35.9, M36.0, M36.1, M36.8, M45.0, M45.1, M45.2, M45.3, M45.4, M45.5, M45.6, M45.7, M45.8, M45.9, M46.00, M46.01, M46.02, M46.03, M46.04, M46.05, M46.06, M46.07, M46.08, M46.09, M46.1, M46.50, M46.51, M46.52, M46.53, M46.54, M46.55, M46.56, M46.57, M46.58, M46.59, M46.80, M46.81, M46.82, M46.83, M46.84, M46.85, M46.86, M46.87, M46.88, M46.89, M46.90, M46.91, M46.92, M46.93, M46.94, M46.95, M46.96, M46.97, M46.98, M46.99, M49.80, M49.81, M49.82, M49.83, M49.84, M49.85, M49.86, M49.87, M49.88, M49.89, 701.0, 710.0, 710.1, 710.2, 710.3, 710.4, 710.5, 710.6, 710.7, 710.8, 710.9, 714.0, 714.1, 714.2, 714.3, 714.4, 714.5, 714.6, 714.7, 714.8, 714.9, 720.0, 720.1, 720.2, 720.3, 720.4, 720.5, 720.6, 720.7, 720.8, 720.9, 725.X
Coagulopathy	D61.09, D61.1, D61.2, D61.3, D61.810, D61.811, D61.818, D61.82, D61.89, D61.9, D65.X, D66.X, D67.X, D68.0, D68.1, D68.2, D68.311, D68.312, D68.318, D68.32, D68.4, D68.8, D68.9, D69.1, D69.3, D69.41, D69.42, D69.49, D69.51, D69.59, D69.6, D69.8, D69.9, D75.82, O99.111, O99.112, O99.113, O99.119, O99.12, O99.13, 286.0, 286.1, 286.2, 286.3, 286.4, 286.5, 286.6, 286.7, 286.8, 286.9, 287.1, 287.3, 287.4, 287.5, 289.84, 649.30, 649.31, 649.32, 649.33, 649.34
Obesity	E66.01, E66.09, E66.1, E66.2, E66.8, E66.9, O99.210, O99.211, O99.212, O99.213, O99.214, O99.215, R93.9, Z68.30, Z68.31, Z68.32, Z68.33, Z68.34, Z68.35, Z68.36, Z68.37, Z68.38, Z68.39, Z68.41, Z68.42, Z68.43, Z68.44, Z68.45, Z68.54, 278.0, 278.00, 278.01, 278.03, 649.10, 649.11, 649.12, 649.13, 649.14, 793.91, V85.30, V85.31, V85.32, V85.33, V85.34, V85.35, V85.36, V85.37, V85.38, V85.39, V85.41, V85.42, V85.43, V85.44, V85.45, V85.54
Weight loss	E40.X, E41.X, E42.X, E43.X, E44.0, E44.1, E45.X, E46.X, E64.0, O25.10, O25.11, O25.12, O25.13, O25.2, O25.3, R63.4, R64.X, 260.X, 261.X, 262.X, 263.X, 260.1, 260.2, 260.3, 260.4, 260.5, 260.6, 260.7, 260.8, 260.9, 261.0, 261.1, 261.2, 261.3, 261.4, 261.5, 261.6, 261.7, 261.8, 261.9, 262.0, 262.1, 262.2, 262.3, 262.4, 262.5, 262.6, 262.7, 262.8, 262.9, 263.0, 263.1, 263.2, 263.3, 263.4, 263.5, 263.6, 263.7, 263.8, 263.9, 783.21, 783.22
Blood loss anemia	D50.0, O90.81, O99.02, O99.03, 280.0, 648.20, 648.21, 648.22, 648.23, 648.24
Deficiency anemia	D50.1, D50.8, D50.9, D51.0, D51.1, D51.2, D51.3, D51.8, D51.9, D52.0, D52.1, D52.8, D52.9, D53.0, D53.1, D53.2, D53.8, D53.9, D63.0, D63.1, D63.8, D64.9, O99.011, O99.012, O99.013, O99.019, 280.1, 280.2, 280.3, 280.4, 280.5, 280.6, 280.7, 280.8, 280.9, 281.0,

© 2022 Lapa ME et al. *JAMA Network Open.*

	281.1, 281.2, 281.3, 281.4, 281.5, 281.6, 281.7, 281.8, 281.9, 285.21, 285.22, 285.23, 285.24, 285.25, 285.26, 285.27, 285.28, 285.29, 285.9
Alcohol abuse	F10.10, F10.11, F10.120, F10.121, F10.129, F10.130, F10.131, F10.132, F10.139, F10.14, F10.150, F10.151, F10.159, F10.180, F10.181, F10.182, F10.188, F10.19, F10.20, F10.21, F10.220, F10.221, F10.229, F10.230, F10.231, F10.232, F10.239, F10.24, F10.250, F10.251, F10.259, F10.26, F10.27, F10.280, F10.281, F10.282, F10.288, F10.29, F10.94, F10.950, F10.951, F10.959, F10.96, F10.97, F10.980, G62.1, I42.6, K29.20, K29.21, K70.10, K70.11, O99.310, O99.311, O99.312, O99.313, O99.314, O99.315, 291.0, 291.1, 291.2, 291.3, 291.5, 291.8, 291.81, 291.82, 291.89, 291.9, 303.00, 303.01, 303.02, 303.03, 303.04, 303.05, 303.06, 303.07, 303.08, 303.09, 303.10, 303.11, 303.12, 303.13, 303.14, 303.15, 303.16, 303.17, 303.18, 303.19, 303.20, 303.21, 303.22, 303.23, 303.24, 303.25, 303.26, 303.27, 303.28, 303.29, 303.30, 303.31, 303.32, 303.33, 303.34, 303.35, 303.36, 303.37, 303.38, 303.39, 303.40, 303.41, 303.42, 303.43, 303.44, 303.45, 303.46, 303.47, 303.48, 303.49, 303.50, 303.51, 303.52, 303.53, 303.54, 303.55, 303.56, 303.57, 303.58, 303.59, 303.60, 303.61, 303.62, 303.63, 303.64, 303.65, 303.66, 303.67, 303.68, 303.69, 303.70, 303.71, 303.72, 303.73, 303.74, 303.75, 303.76, 303.77, 303.78, 303.79, 303.80, 303.81, 303.82, 303.83, 303.84, 303.85, 303.86, 303.87, 303.88, 303.89, 303.90, 303.91, 303.92, 303.93, 305.00, 305.01, 305.02, 305.03
Drug abuse	F11.10, F11.11, F11.120, F11.121, F11.122, F11.129, F11.13, F11.14, F11.150, F11.151, F11.159, F11.181, F11.182, F11.188, F11.19, F11.20, F11.21, F11.220, F11.221, F11.222, F11.229, F11.23, F11.24, F11.250, F11.251, F11.259, F11.281, F11.282, F11.288, F11.29, F12.10, F12.11, F12.120, F12.121, F12.122, F12.129, F12.13, F12.150, F12.151, F12.159, F12.180, F12.188, F12.19, F12.20, F12.21, F12.220, F12.221, F12.222, F12.229, F12.23, F12.250, F12.251, F12.259, F12.280, F12.288, F12.29, F13.10, F13.11, F13.120, F13.121, F13.129, F13.130, F13.131, F13.132, F13.139, F13.14, F13.150, F13.151, F13.159, F13.180, F13.181, F13.182, F13.188, F13.19, F13.20, F13.21, F13.220, F13.221, F13.229, F13.230, F13.231, F13.232, F13.239, F13.24, F13.250, F13.251, F13.259, F13.26, F13.27, F13.280, F13.281, F13.282, F13.288, F13.29, F14.10, F14.11, F14.120, F14.121, F14.122, F14.129, F14.13, F14.14, F14.150, F14.151, F14.159, F14.180, F14.181, F14.182, F14.188, F14.19, F14.20, F14.21, F14.220, F14.221, F14.222, F14.229, F14.23, F14.24, F14.250, F14.251, F14.259, F14.280, F14.281, F14.282, F14.288, F14.29, F15.10, F15.11, F15.120, F15.121, F15.122, F15.129, F15.13, F15.14, F15.150, F15.151, F15.159, F15.180, F15.181, F15.182, F15.188, F15.19, F15.20, F15.21, F15.220, F15.221, F15.222, F15.229, F15.23, F15.24, F15.250, F15.251, F15.259, F15.280, F15.281, F15.282, F15.288, F15.29, F16.10, F16.11, F16.120, F16.121, F16.122, F16.129, F16.14, F16.150, F16.151, F16.159, F16.180, F16.183, F16.188, F16.19, F16.20, F16.21, F16.220, F16.221, F16.229, F16.24, F16.250, F16.251, F16.259, F16.280, F16.283, F16.288, F16.29, F18.10, F18.11, F18.120, F18.121, F18.129, F18.14, F18.150, F18.151, F18.159, F18.17, F18.180, F18.188, F18.19, F18.20, F18.21, F18.220, F18.221, F18.229, F18.24, F18.250, F18.251, F18.259, F18.27, F18.280, F18.288, F18.29, F19.10, F19.11, F19.120, F19.121, F19.122, F19.129, F19.130, F19.131, F19.132, F19.139, F19.14, F19.150, F19.151, F19.159, F19.16, F19.17, F19.180, F19.181, F19.182, F19.188, F19.19, F19.20, F19.21, F19.220, F19.221, F19.222, F19.229, F19.230, F19.231, F19.232, F19.239, F19.24, F19.250, F19.251, F19.259, F19.26, F19.27, F19.280, F19.281, F19.282, F19.288, F19.29, O99.320, O99.321, O99.322, O99.323, O99.324, O99.325, 292.0, 292.82, 292.89, 292.9, 304.00, 304.01, 304.02, 304.03, 304.04, 304.05, 304.06, 304.07, 304.08, 304.09, 304.10, 304.11, 304.12, 304.13, 304.14, 304.15, 304.16,

© 2022 Lapa ME et al. *JAMA Network Open.*

	304.17, 304.18, 304.19, 304.20, 304.21, 304.22, 304.23, 304.24, 304.25, 304.26, 304.27, 304.28, 304.29, 304.30, 304.31, 304.32, 304.33, 304.34, 304.35, 304.36, 304.37, 304.38, 304.39, 304.40, 304.41, 304.42, 304.43, 304.44, 304.45, 304.46, 304.47, 304.48, 304.49, 304.50, 304.51, 304.52, 304.53, 304.54, 304.55, 304.56, 304.57, 304.58, 304.59, 304.60, 304.61, 304.62, 304.63, 304.64, 304.65, 304.66, 304.67, 304.68, 304.69, 304.70, 304.71, 304.72, 304.73, 304.74, 304.75, 304.76, 304.77, 304.78, 304.79, 304.80, 304.81, 304.82, 304.83, 304.84, 304.85, 304.86, 304.87, 304.88, 304.89, 304.90, 304.91, 304.92, 304.93, 305.20, 305.21, 305.22, 305.23, 305.24, 305.25, 305.26, 305.27, 305.28, 305.29, 305.30, 305.31, 305.32, 305.33, 305.34, 305.35, 305.36, 305.37, 305.38, 305.39, 305.40, 305.41, 305.42, 305.43, 305.44, 305.45, 305.46, 305.47, 305.48, 305.49, 305.50, 305.51, 305.52, 305.53, 305.54, 305.55, 305.56, 305.57, 305.58, 305.59, 305.60, 305.61, 305.62, 305.63, 305.64, 305.65, 305.66, 305.67, 305.68, 305.69, 305.70, 305.71, 305.72, 305.73, 305.74, 305.75, 305.76, 305.77, 305.78, 305.79, 305.80, 305.81, 305.82, 305.83, 305.84, 305.85, 305.86, 305.87, 305.88, 305.89, 305.90, 305.91, 305.92, 305.93, 648.30, 648.31, 648.32, 648.33, 648.34
Psychoses	F06.0, F06.1, F06.2, F06.30, F06.33, F11.150, F11.151, F11.159, F11.250, F11.251, F11.259, F11.950, F11.951, F11.959, F12.150, F12.151, F12.159, F12.250, F12.251, F12.259, F12.950, F12.951, F12.959, F13.150, F13.151, F13.159, F13.250, F13.251, F13.259, F13.950, F13.951, F13.959, F14.150, F14.151, F14.159, F14.250, F14.251, F14.259, F14.950, F14.951, F14.959, F15.150, F15.151, F15.159, F15.250, F15.251, F15.259, F15.950, F15.951, F15.959, F16.150, F16.151, F16.159, F16.250, F16.251, F16.259, F16.950, F16.951, F16.959, F18.150, F18.151, F18.159, F18.250, F18.251, F18.259, F18.950, F18.951, F18.959, F19.150, F19.151, F19.159, F19.250, F19.251, F19.259, F19.950, F19.951, F19.959, F20.0, F20.1, F20.2, F20.3, F20.5, F20.81, F20.89, F20.9, F21.X, F22.X, F23.X, F24.X, F25.0, F25.1, F25.8, F25.9, F28.X, F29.X, F30.10, F30.11, F30.12, F30.13, F30.2, F30.3, F30.4, F30.8, F30.9, F31.0, F31.10, F31.11, F31.12, F31.13, F31.2, F31.30, F31.31, F31.32, F31.4, F31.5, F31.60, F31.61, F31.62, F31.63, F31.64, F31.70, F31.71, F31.72, F31.73, F31.74, F31.75, F31.76, F31.77, F31.78, F31.81, F31.89, F31.9, F32.4, F32.5, F33.40, F33.41, F33.42, F34.0, F34.8, F34.81, F34.89, F34.9, F39.X, F44.89, F84.3, 295.00, 295.01, 295.02, 295.03, 295.04, 295.05, 295.06, 295.07, 295.08, 295.09, 295.10, 295.11, 295.12, 295.13, 295.14, 295.15, 295.16, 295.17, 295.18, 295.19, 295.20, 295.21, 295.22, 295.23, 295.24, 295.25, 295.26, 295.27, 295.28, 295.29, 295.30, 295.31, 295.32, 295.33, 295.34, 295.35, 295.36, 295.37, 295.38, 295.39, 295.40, 295.41, 295.42, 295.43, 295.44, 295.45, 295.46, 295.47, 295.48, 295.49, 295.50, 295.51, 295.52, 295.53, 295.54, 295.55, 295.56, 295.57, 295.58, 295.59, 295.60, 295.61, 295.62, 295.63, 295.64, 295.65, 295.66, 295.67, 295.68, 295.69, 295.70, 295.71, 295.72, 295.73, 295.74, 295.75, 295.76, 295.77, 295.78, 295.79, 295.80, 295.81, 295.82, 295.83, 295.84, 295.85, 295.86, 295.87, 295.88, 295.89, 295.90, 295.91, 295.92, 295.93, 295.94, 295.95, 295.96, 295.97, 295.98, 295.99, 296.00, 296.01, 296.02, 296.03, 296.04, 296.05, 296.06, 296.07, 296.08, 296.09, 296.10, 296.11, 296.12, 296.13, 296.14, 296.15, 296.16, 296.17, 296.18, 296.19, 296.20, 296.21, 296.22, 296.23, 296.24, 296.25, 296.26, 296.27, 296.28, 296.29, 296.30, 296.31, 296.32, 296.33, 296.34, 296.35, 296.36, 296.37, 296.38, 296.39, 296.40, 296.41, 296.42, 296.43, 296.44, 296.45, 296.46, 296.47, 296.48, 296.49, 296.50, 296.51, 296.52, 296.53, 296.54, 296.55, 296.56, 296.57, 296.58, 296.59, 296.60, 296.61, 296.62, 296.63, 296.64, 296.65, 296.66, 296.67, 296.68, 296.69, 296.70, 296.71, 296.72, 296.73, 296.74, 296.75, 296.76, 296.77, 296.78, 296.79, 296.80, 296.81, 296.82, 296.83,

© 2022 Lapa ME et al. *JAMA Network Open.*

	296.84, 296.85, 296.86, 296.87, 296.88, 296.89, 296.90, 296.91, 296.92, 296.93, 296.94, 296.95, 296.96, 296.97, 296.98, 296.99, 297.00, 297.01, 297.02, 297.03, 297.04, 297.05, 297.06, 297.07, 297.08, 297.09, 297.10, 297.11, 297.12, 297.13, 297.14, 297.15, 297.16, 297.17, 297.18, 297.19, 297.20, 297.21, 297.22, 297.23, 297.24, 297.25, 297.26, 297.27, 297.28, 297.29, 297.30, 297.31, 297.32, 297.33, 297.34, 297.35, 297.36, 297.37, 297.38, 297.39, 297.40, 297.41, 297.42, 297.43, 297.44, 297.45, 297.46, 297.47, 297.48, 297.49, 297.50, 297.51, 297.52, 297.53, 297.54, 297.55, 297.56, 297.57, 297.58, 297.59, 297.60, 297.61, 297.62, 297.63, 297.64, 297.65, 297.66, 297.67, 297.68, 297.69, 297.70, 297.71, 297.72, 297.73, 297.74, 297.75, 297.76, 297.77, 297.78, 297.79, 297.80, 297.81, 297.82, 297.83, 297.84, 297.85, 297.86, 297.87, 297.88, 297.89, 297.90, 297.91, 297.92, 297.93, 297.94, 297.95, 297.96, 297.97, 297.98, 297.99, 298.00, 298.01, 298.02, 298.03, 298.04, 298.05, 298.06, 298.07, 298.08, 298.09, 298.10, 298.11, 298.12, 298.13, 298.14, 298.15, 298.16, 298.17, 298.18, 298.19, 298.20, 298.21, 298.22, 298.23, 298.24, 298.25, 298.26, 298.27, 298.28, 298.29, 298.30, 298.31, 298.32, 298.33, 298.34, 298.35, 298.36, 298.37, 298.38, 298.39, 298.40, 298.41, 298.42, 298.43, 298.44, 298.45, 298.46, 298.47, 298.48, 298.49, 298.50, 298.51, 298.52, 298.53, 298.54, 298.55, 298.56, 298.57, 298.58, 298.59, 298.60, 298.61, 298.62, 298.63, 298.64, 298.65, 298.66, 298.67, 298.68, 298.69, 298.70, 298.71, 298.72, 298.73, 298.74, 298.75, 298.76, 298.77, 298.78, 298.79, 298.80, 298.81, 298.82, 298.83, 298.84, 298.85, 298.86, 298.87, 298.88, 298.89, 298.9, 298.90, 299.10, 299.11
Depression	F06.31, F06.32, F06.34, F32.0, F32.1, F32.2, F32.3, F32.8, F32.81, F32.89, F32.9, F33.0, F33.1, F33.2, F33.3, F33.8, F33.9, F34.1, 300.4, 301.12, 309.0, 309.1, 311.X
Cerebrovascular disease	G45.0, G45.1, G45.2, G45.3, G45.4, G45.8, G45.9, G46.0, G46.1, G46.2, G46.3, G46.4, G46.5, G46.6, G46.7, G46.8, H34.00, H34.01, H34.02, H34.03, H34.10, H34.11, H34.12, H34.13, H34.211, H34.212, H34.213, H34.219, H34.231, H34.232, H34.233, H34.239, I60.00, I60.01, I60.02, I60.10, I60.11, I60.12, I60.2, I60.20, I60.21, I60.22, I60.30, I60.31, I60.32, I60.4, I60.50, I60.51, I60.52, I60.6, I60.7, I60.8, I60.9, I61.0, I61.1, I61.2, I61.3, I61.4, I61.5, I61.6, I61.8, I61.9, I62.00, I62.03, I62.1, I62.9, I63.00, I63.011, I63.012, I63.013, I63.019, I63.02, I63.031, I63.032, I63.033, I63.039, I63.09, I63.10, I63.111, I63.112, I63.113, I63.119, I63.12, I63.131, I63.132, I63.133, I63.139, I63.19, I63.20, I63.211, I63.212, I63.213, I63.219, I63.22, I63.231, I63.232, I63.233, I63.239, I63.29, I63.30, I63.311, I63.312, I63.313, I63.319, I63.321, I63.322, I63.323, I63.329, I63.331, I63.332, I63.333, I63.339, I63.341, I63.342, I63.343, I63.349, I63.39, I63.40, I63.411, I63.412, I63.413, I63.419, I63.421, I63.422, I63.423, I63.429, I63.431, I63.432, I63.433, I63.439, I63.441, I63.442, I63.443, I63.449, I63.49, I63.50, I63.511, I63.512, I63.513, I63.519, I63.521, I63.522, I63.523, I63.529, I63.531, I63.532, I63.533, I63.539, I63.541, I63.542, I63.543, I63.549, I63.59, I63.6, I63.8, I63.81, I63.89, I63.9, I65.01, I65.02, I65.03, I65.09, I65.1, I65.21, I65.22, I65.23, I65.29, I65.8, I65.9, I66.01, I66.02, I66.03, I66.09, I66.11, I66.12, I66.13, I66.19, I66.21, I66.22, I66.23, I66.29, I66.3, I66.8, I66.9
Cerebrovascular disease sequelae	I69.30, I69.31, I69.310, I69.311, I69.312, I69.313, I69.314, I69.315, I69.318, I69.319, I69.320, I69.321, I69.322, I69.323, I69.328, I69.331, I69.332, I69.333, I69.334, I69.339, I69.341, I69.342, I69.343, I69.344, I69.349, I69.351, I69.352, I69.353, I69.354, I69.359, I69.361, I69.362, I69.363, I69.364, I69.365, I69.369, I69.390, I69.391, I69.392, I69.393, I69.398, I69.80, I69.81, I69.810, I69.811, I69.812, I69.813, I69.814, I69.815, I69.818, I69.819, I69.820, I69.821, I69.822, I69.823, I69.828, I69.831, I69.832, I69.833, I69.834,

© 2022 Lapa ME et al. *JAMA Network Open.*

	I69.839, I69.841, I69.842, I69.843, I69.844, I69.849, I69.851, I69.852, I69.853, I69.854, I69.859, I69.861, I69.862, I69.863, I69.864, I69.865, I69.869, I69.890, I69.891, I69.892, I69.893, I69.898, I69.90, I69.91, I69.910, I69.911, I69.912, I69.913, I69.914, I69.915, I69.918, I69.919, I69.920, I69.921, I69.922, I69.923, I69.928, I69.931, I69.932, I69.933, I69.934, I69.939, I69.941, I69.942, I69.943, I69.944, I69.949, I69.951, I69.952, I69.953, I69.954, I69.959, I69.961, I69.962, I69.963, I69.964, I69.965, I69.969, I69.990, I69.991, I69.992, I69.993, I69.998, P91.821, P91.822, P91.823, P91.829
Other thyroid disorders	E04.0, E04.1, E04.2, E04.8, E04.9, E05.00, E05.01, E05.10, E05.11, E05.20, E05.21, E05.30, E05.31, E05.40, E05.41, E05.80, E05.81, E05.90, E05.91, E06.0, E06.1, E06.2, E06.3, E06.4, E06.5, E06.9, O90.5
Dementia	F01.50, F01.51, F02.80, F02.81, F03.90, F03.91, G30.0, G30.1, G30.8, G30.9, G31.01, G31.09, G31.1, G31.2, G31.81, G31.82, G31.83, G31.85, G31.89, G31.9
Bipolar Disorder	F3010, F3011, F3012, F3013, F302, F303, F304, F308, F309, F310, F3110, F3111, F3112, F3113, F312, F3130, F3131, F3132, F314, F315, F3160, F3161, F3162, F3163, F3164, F3170, F3171, F3172, F3173, F3174, F3175, F3176, F3177, F3178, F3181, F3189, F319, F338, F3481, F3489, F349, F39, 296.00, 296.01, 296.02, 296.03, 296.04, 296.05, 296.06, 296.10, 296.11, 296.12, 296.13, 296.14, 296.15, 296.16, 296.40, 296.41, 296.42, 296.43, 296.44, 296.45, 296.46, 296.50, 296.51, 296.52, 296.53, 296.54, 296.55, 296.56, 296.60, 296.61, 296.62, 296.63, 296.64, 296.65, 296.66, 296.70, 296.80, 296.81, 296.82, 296.89, 296.90, 2969.9
Schizophrenia	F060, F062, F200, F201, F202, F203, F205, F2081, F2089, F209, F250, F251, F258, F259, 295.00, 295.01, 295.02, 295.03, 295.04, 295.05, 295.10, 295.11, 295.12, 295.13, 295.14, 295.15, 295.20, 295.21, 295.22, 295.23, 295.24, 295.25, 295.30, 295.31, 295.32, 295.33, 295.34, 295.35, 295.40, 295.41, 295.42, 295.43, 295.44, 295.45, 295.50, 295.51, 295.52, 295.53, 295.54, 295.55, 295.60, 295.61, 295.62, 295.63, 295.64, 295.65, 295.70, 295.71, 295.72, 295.73, 295.74, 295.75, 295.80, 295.81, 295.82, 295.83, 295.84, 295.85, 295.90, 295.91, 295.92, 295.93, 295.94, 295.95
Personality Disorder	F21, F340, F341, F600, F601, F602, F603, F604, F605, F606, F607, F6081, F6089, F609, F6810, F6811, F6812, F6813, F69, 301.0, 301.10, 301.11, 301.12, 301.13, 30.120, 301.21, 301.22, 301.3, 301.4, 301.50, 301.51, 301.59, 301.6, 301.7, 301.81, 301.82, 301.83, 301.84, 301.89, 301.9
Anxiety Disorder	F064, F4000, F4001, F4002, F4010, F4011, F40210, F40218, F40220, F40228, F40230, F40231, F40232, F40233, F40240, F40241, F40242, F40243, F40248, F40290, F40291, F40298, F408, F409, F410, F411, F413, F418, F419, F42, F422, F423, F424, F428, F429, F430, F4310, F4311, F4312, F449, F458, F488, F489, F938, F99, R452, R455, R456, R457, 293.84, 300.00, 300.01, 300.02, 300.09, 300.10, 300,20, 300.21, 300.22, 30023, 300.29, 300.3, 300.5, 300.89, 300.9, 308.0, 308.1, 308.2, 308.3, 308.4, 308.9, 309.81, 313.0, 313.1, 313.21, 313.22, 313.3, 313.82, 313.83
Post-Traumatic Stress Disorder	F4310, F4311, F4312, 30981
Past acute myocardial infarction	I21.0, I21.1, I21.2, I21.3, I21.4, I21.9, I22.X, 410.X

© 2022 Lapa ME et al. *JAMA Network Open.*

Procedure	Procedure Code (CPT)
Percutaneous coronary intervention	92920, 92921, 92924, 92925, 92928, 92929, 92933, 92934, 92937, 92938, 92941, 92943, 92944, 92973, 92974
ICD-9-CM and ICD-10-CM indicates International Classification of Diseases, Ninth and Tenth Revisions, Clinical Modification codes. CPT indicates Current Procedural Terminology, Version 4 procedure codes.	

© 2022 Lapa ME et al. *JAMA Network Open.*

eTable 2. Medications and Respective Classes Used in Analysis	
**Medication Class**	**Medications**
Antiplatelets	Clopidogrel, Ticagrelor, Prasugrel
Beta Blockers	Atenolol, Bisoprolol, Carvedilol, Labetalol, Metoprolol, Propranolol, Sotalol, Nadolol, Nebivolol
ACE-inhibitors	Benazepril, Captopril, Enalapril, Fosinopril, Lisinopril, Quinapril, Ramipril
ARBs	Valsartan, Losartan, Olmesartan, Telmisartan, Candesartan
Statins	Atorvastatin, Pravastatin, Rosuvastatin, Simvastatin, Fluvastatin, Lovastatin
Antidepressants	Selective Serotonin Reuptake Inhibitors: Sertraline, Citalopram, Escitalopram, Fluoxetine, Paroxetine<br>Serotonin-Norepinephrine Reuptake Inhibitors: Duloxetine, Venlafaxine
ACE-inhibitors indicates angiotensin converting enzyme inhibitors; ARBs, aldosterone receptor blockers.	

© 2022 Lapa ME et al. *JAMA Network Open.*

eTable 3. Sensitivity Analyses for Adherence to Guideline-Directed Medical Therapies Following Coronary Intervention in Individuals With Depression Compared With Those Without Depression

	Adequate Adherence (PDC ≥80 to <90%)					
	Model 3A[a]		Model 3B[b]		Model 3C[c]	
**GDMT Medication Class**	**OR (95% CI)**	**p-value**	**OR (95% CI)**	**p-value**	**OR (95% CI)**	**p-value**
Antiplatelets	0.79 (0.73, 0.85)	<.0001	0.85 (0.80, 0.90)	<.0001	0.80 (0.75, 0.86)	<.0001
Beta Blockers	0.80 (0.75, 0.85)	<.0001	0.90 (0.85, 0.95)	.0001	0.83 (0.76, 0.90)	<.0001
RAAS Inhibitors	0.90 (0.80, 1.02)	.09	0.93 (0.85, 1.03)	.17	0.97 (0.83, 1.13)	.69
Statins	0.87 (0.81, 0.93)	<.0001	0.93 (0.88, 0.98)	.009	0.84 (0.76, 0.92)	.0002
	**Optimal Adherence (PDC ≥90%)**					
	**Model 3A[a]**		**Model 3B[b]**		**Model 3C[c]**	
**GDMT Medication Class**	**OR (95% CI)**	**p-value**	**OR (95% CI)**	**p-value**	**OR (95% CI)**	**p-value**
Antiplatelets	0.77 (0.73, 0.82)	<.0001	0.83 (0.79, 0.87)	<.0001	0.79 (0.75, 0.84)	<.0001
Beta Blockers	0.76 (0.72, 0.81)	<.0001	0.83 (0.79, 0.87)	<.0001	0.74 (0.68, 0.79)	<.0001
RAAS Inhibitors	0.83 (0.75, 0.92)	.0003	0.89 (0.82, 0.97)	.006	0.89 (0.78, 1.02)	.10
Statins	0.81 (0.77, 0.86)	<.0001	0.87 (0.83, 0.91)	<.0001	0.82 (0.75, 0.89)	<.0001

PDC indicates Proportion of Days Covered; RAAS inhibitors, renin-angiotensin-aldosterone system inhibitors (angiotensin converting enzyme inhibitors and angiotensin receptor blockers). All models adjusted for age sex, race, number of medical comorbidities, comorbid psychiatric conditions, educational attainment, income level, insurance type, number of medications used, and switching medication during follow-up.

[a] Model 3A – excluding individuals with psychiatric diagnoses (anxiety disorder, bipolar disorder, personality disorder, post-traumatic stress disorder, or schizophrenia).

[b] Model 3B – depression status classified by diagnosis and use of selective serotonin reuptake inhibitor or serotonin and norepinephrine reuptake inhibitor.

[c] Model 3C – excluding individuals with prior history of GDMT agent use.

© 2022 Lapa ME et al. *JAMA Network Open.*

eTable 4. Sensitivity Analysis for Adherence (Adequate and Optimal) to Guideline-Directed Medical Therapies Following Coronary Intervention in Individuals With Depression Compared With Those Without Depression

	**Adequate Adherence (PDC ≥80 to <90%) or Optimal Adherence (PDC ≥ 90%)**				
			**Model 3[a]**		
**Medication Class**			**OR (95% CI)**	**p-value**	
**Antiplatelets**			0.82 (0.78, 0.87)	<.0001	
**Beta Blockers**			0.84 (0.80, 0.88)	<.0001	
**RAAS Inhibitors**			0.93 (0.85, 1.01)	.073	
**Statins**			0.89 (0.84, 0.93)	<.0001	

PDC indicates Proportion of Days Covered; RAAS inhibitors, renin-angiotensin-aldosterone system inhibitors (angiotensin converting enzyme inhibitors and angiotensin receptor blockers).
[a]Model 3 – adjusted for age sex, race, number of medical comorbidities, comorbid psychiatric conditions, educational attainment, income level, insurance type, number of medications used, and switching medication during follow-up.

eFigure. Cohort Selection Flow Chart

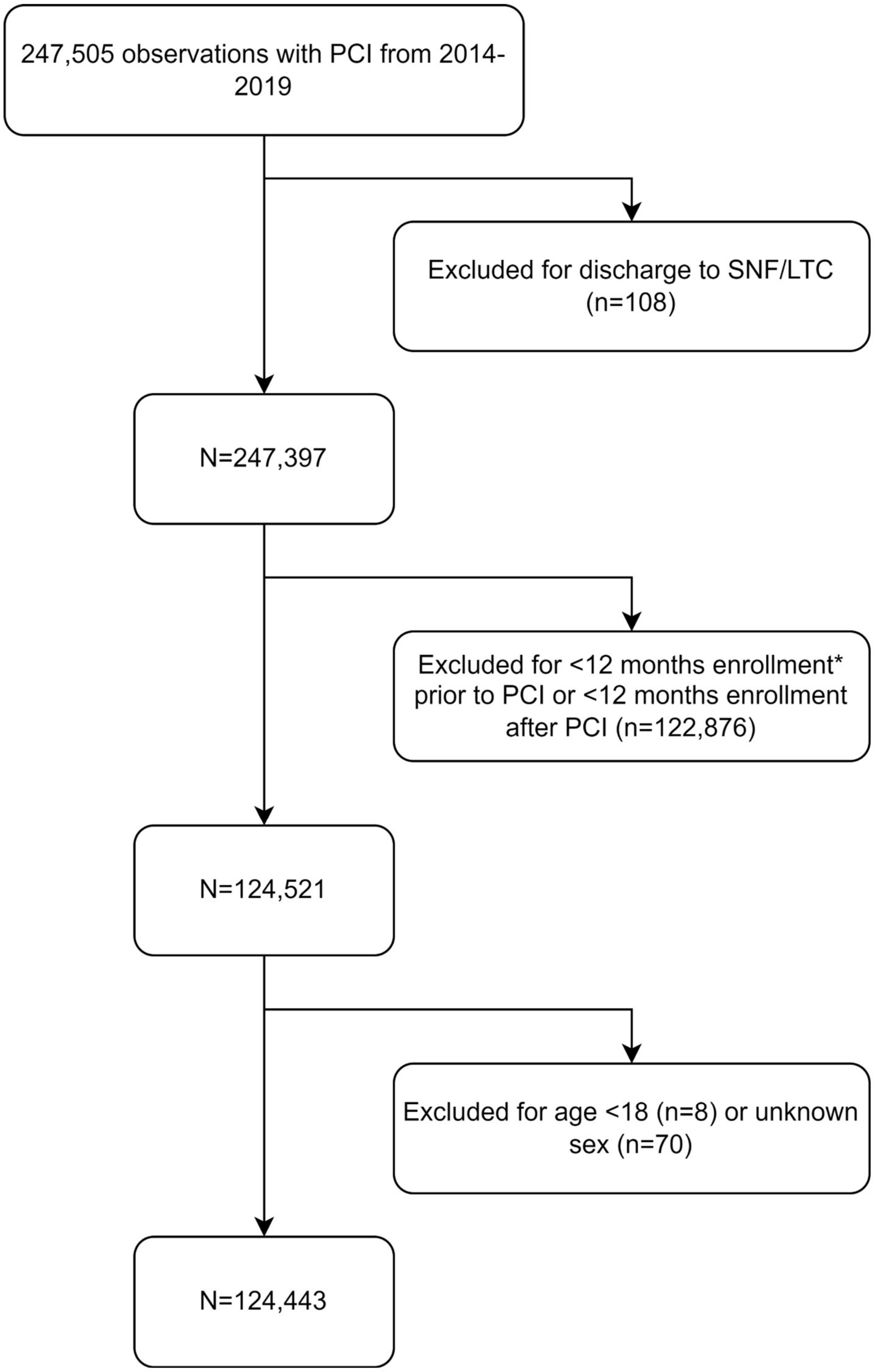

Flow chart of the selection of individuals within the cohort. There were 247,505 individuals identified who underwent percutaneous coronary intervention (PCI) between January 1, 2014, to December 31, 2019. Individuals were excluded if they were discharged from PCI to long-term care center or skilled-nursing facility, less than 12 months of enrollment prior PCI and 12 months afterwards, age <18, and those with missing sex. After exclusions 124,443 individuals were eligible for analysis.

© 2022 Lapa ME et al. *JAMA Network Open.*
